# Tracing the molecular basis of transcriptional dynamics in noisy data by using an experiment-based mathematical model

**DOI:** 10.1093/nar/gku1272

**Published:** 2014-12-03

**Authors:** Katja N. Rybakova, Aleksandra Tomaszewska, Simon van Mourik, Joke Blom, Hans V. Westerhoff, Carsten Carlberg, Frank J. Bruggeman

**Affiliations:** 1 Molecular Cell Physiology, VU University Amsterdam, De Boelelaan 1087, NL-1081 HV Amsterdam, The Netherlands; 2 School of Medicine, Institute of Biomedicine, University of Eastern Finland, FI-70211 Kuopio, Finland; 3 Biometris, Plant Sciences Group, Wageningen University and Research Center, NL-6708 PB Wageningen, The Netherlands; 4 Life Sciences, Centre for Mathematics and Computer Science (CWI), NL-1098 XG Amsterdam, The Netherlands; 5 Manchester Centre for Integrative Systems Biology, CEAS, University of Manchester, Manchester M60 1QD, UK; 6 Synthetic Systems Biology, Swammerdam Institute for Life Sciences, University of Amsterdam, NL-1098 XH Amsterdam, The Netherlands; 7 Systems Bioinformatics, VU University Amsterdam, De Boelelaan 1087, NL-1081 HV Amsterdam, The Netherlands

## Abstract

Changes in transcription factor levels, epigenetic status, splicing kinetics and mRNA degradation can each contribute to changes in the mRNA dynamics of a gene. We present a novel method to identify which of these processes is changed in cells in response to external signals or as a result of a diseased state. The method employs a mathematical model, for which the kinetics of gene regulation, splicing, elongation and mRNA degradation were estimated from experimental data of transcriptional dynamics. The time-dependent dynamics of several species of adipose differentiation-related protein (ADRP) mRNA were measured in response to ligand activation of the transcription factor peroxisome proliferator-activated receptor δ (PPARδ). We validated the method by monitoring the mRNA dynamics upon gene activation in the presence of a splicing inhibitor. Our mathematical model correctly identifies splicing as the inhibitor target, despite the noise in the data.

## INTRODUCTION

The production and processing of mRNA is a highly regulated and dynamic process, which is often disturbed in disease (1–3). Deep-sequencing technologies enable high-resolution quantitation and monitoring of RNA species, such as splicing variants, small non-coding RNAs and intermediates in mRNA synthesis for all expressed genes of a cell (4–6). RNA datasets obtained under various conditions, such as perturbation of cells with signaling molecules, may be compared. However, it remains hard to infer from differences between such RNA datasets which particular molecular process is the key determinant of the alterations in mRNA dynamics (7–9). An interesting challenge to systems biology approaches is therefore to develop methods that infer molecular causes from such datasets. This may also enable the identification of the dominant perturbed processes in cases of disease.

An important tool in this context is the ability to measure mRNA dynamics in cultured cells, which has led to many new observations, such as transcriptional cycling—oscillatory changes in mRNA levels linked to periodic switching of gene promoters (10,11). However, the interpretation of such observations and the elucidation of mechanisms underpinning them are greatly hampered by the noise in the data (12,13). Mathematical models can be very informative in such noisy contexts (14,15). Such models have the advantage that they enable a more objective evaluation of the conclusions that can be drawn directly from the data. This is even more important in cases when data are potentially oscillatory but also have high measurement noise, when an intuitive interpretation is insufficient.

Mathematical methods for model development on the basis of experimental data, such as parameter estimation, model discrimination and experimental design, offer a rigorous methodology to integrate experimental data on molecular concentrations with molecular network architecture (16–19). These methods facilitate the development of rational perturbation strategies and the identification of perturbation targets. They likewise improve the understanding of signaling, metabolic and gene networks (20–23). In this study, we used these techniques to develop a methodology for modeling that enables inference of the kinetics of the molecular processes underlying mRNA dynamics. We identify the perturbed molecular processes from a comparison of mRNA time-series in unperturbed in comparison to perturbed cells.

The human *ADRP* gene is a direct target of the transcription factor PPARδ (24–27). The function of the ADRP protein (also known as PLIN2 or ADFP) is associated with lipid accumulation and lipid droplet formation (26). The *ADRP* gene was initially assumed to be specific to adipocytes and their differentiation process, but now it is clear that the gene is expressed in all metabolic tissues (28). Because nuclear receptors, such as PPARδ, are directly activated by the binding of their cognate ligand, confounding effects of signaling pathways on mRNA dynamics of their primary target genes are limited (29). Therefore, the regulation of the *ADRP* gene in response to PPARδ activation is an attractive system to study temporal transcription-activation responses.

In this study, we obtained experimental data concerning mRNA dynamics of the *ADRP* gene in HepG2 human liver cells. By the use of mathematical modeling, we demonstrated that we are able to treat the data objectively by comparing the prediction of a number of well-defined models with the noisy experimental data and then obtaining best fits. The best fits then lead to the rejection of some models, the choice of a suitable model and estimates of parameters that are also otherwise important for the understanding of cell biology. The model we considered incorporates fundamental processes of mRNA synthesis and processing, such as the promoter ON and OFF cycle, elongation, splicing, maturation and degradation. Model parameters were estimated by fitting model predictions to our experimental time courses of the concentrations of RNA species. We measured an additional time course in the presence of a splicing inhibitor. By re-fitting of the model, we identified the parameter corresponding to splicing process as the most likely target of the perturbation. In conclusion, this study demonstrates how mathematical modeling of transcription can help to identify which molecular processes are perturbed in disease or upon treatment with natural or synthetic signaling molecules.

## MATERIALS AND METHODS

### Cell culture

HepG2 human hepatocellular carcinoma cells were cultured in Dulbecco's modified Eagle's medium (DMEM) medium containing 10% fetal bovine serum (FBS), 2 mM glutamine and 100 U/ml of a penicillin–streptomycin mixture in a humidified 95% air/5% CO_2_ incubator. Approximately 24 h prior to treatment cells were seeded into medium with 5% charcoal-treated FBS in culture flasks containing 10^6^ cells. For all experiments, cells were treated either with solvent dimethyl sulfoxide (DMSO) at a final concentration of 0.1% or with 100 nM of the PPARδ ligand GW501516 (Alexis Biochemicals, San Diego, CA, USA).

### RNA extraction and real-time quantitative PCR (qPCR)

Total RNA was extracted from 10^6^ cells using the Quick-RNA™ MiniPrep isolation kit (Zymo Research, Irvine, CA, USA). cDNA synthesis was performed for 60 min at 37°C using 40 U of M-MuLV reverse transcriptase (Thermo Scientific), 1 μg of total RNA as a template and 100 pM oligodT_18_ or random hexamer primers. qPCR was performed on an iCycler (BioRad) using Absolute SYBR Green Fluorescein (Thermo Scientific). The polymerase chain reaction (PCR) conditions were: pre-incubation for 15 min at 95°C, followed by amplification steps of 40 cycles of 30 s at 95°C, 30 s at primer specific temperatures (Supplementary Table S1), 40 s at 72°C and a final elongation process of 5 min at 72°C. The fold induction was calculated using the formula 2^−(ΔΔCt)^, where ΔΔCt is the ΔCt_(ligand)_ − ΔCt_(DMSO)_, ΔCt is the Ct_(*ADRP*)_ − Ct_(_*_RPLP0_*_)_ and Ct is the cycle number at which the threshold is crossed and *RPLP0* the reference gene ribosomal protein, large, P0. Quality of the PCR product was monitored using post-PCR melt curve analysis. The ΔCt_(ligand)_ − ΔCt_(DMSO)_ did not change significantly with duration of the experiment (Supplementary Figure S1).

### RNA copy number measurement

cDNA was amplified by qPCR using *ADRP*-specific primers (Supplementary Table S1), the products were resolved on a 1.5% agarose gel and cleaned using the QIAEX^®^ II gel extraction kit (Qiagen, Hilden, Germany). The DNA amounts were measured using the Quant-iT™ PicoGreen^®^ dsDNA Assay Kit (Invitrogen) and the numbers of *ADRP* fragment copies per unit sample volume were calculated. A series of 1/10 dilutions was used to create a standard curve of the Ct value against copy number of the molecules added to reaction (Supplementary Figure S2A–C). Transcripts of *barnase*-coding polyadenylated mRNA were produced using an *in vitro* transcription kit (Promega). The transcript concentration was measured by Nanodrop. The transcript was added to the cDNA synthesis reaction in the amounts of 6 × 10^7^ to 1.5 × 10^6^ copies and the resulting amount of cDNA was measured according to a Ct versus copy number standard curve (Supplementary Figure S2D), in order to establish the efficiency of cDNA synthesis. This standard curve was used to calculate the number of copies in DMSO-treated samples known to have *ADRP* expression close to average. From this, the copy numbers were calculated in the cDNA solution, the mRNA extract and the cell, taking into account the cell number in the culture (counted before seeding; doubling time of the cells much exceeded than the duration of the experiment) and maximal mRNA yield per culture. An example of calculation is shown in the Supplemental Material). Replicate samples of each data point were corrected for the outliers with Median Absolute Deviation method (30). The significance of mRNA induction in respect to untreated samples was determined by two-tailed, paired Student's *t*-test. Using Mann–Whitney test that does not require the normal distribution of the data did not alter the results.

### RNA degradation

HepG2 cells were treated either with vehicle (DMSO, final concentration 0.1%) or 100 nM GW501516 for 3 h and then new RNA synthesis was blocked by treating the cells with 50 μM 5,6-dichloro-1-β-d-ribofuranosylbenzimidazole (DRB, Sigma–Aldrich).

### Splicing inhibition

HepG2 cells (3× 10^5^) were seeded into medium with 5% charcoal-treated FBS. The splicing inhibitor isoginkgetin (kindly provided by the Dr Willmar, Schwabe GmbH & Co. KG, Karlsruhe, Germany) was added after 24 h to a final concentration of 50 μM. Sixteen hours later cells were treated either with solvent (DMSO, final concentration 0.1%) or ligand GW501516 (final concentration 100 nM).

### Modeling and parameter fitting

The general models of mRNA metabolism were implemented and the time courses were simulated in Mathematica 8.0 (Wolfram Research Inc., Champaign, IL, USA) using standard inbuilt algorithms. The time course data was fitted using a customary algorithm programmed in Matlab (The MathWorks, Inc., Natick, MA, USA). *A posteriori* identification analysis and additional data fitting was carried out in Mathematica using a Differential Evolution method of the NMinimize algorithm. Details of the system structure parameters and approaches are given in the Supplemental Material.

## RESULTS

### Model of transcriptional dynamics and experimental approach

**Figure 1.**
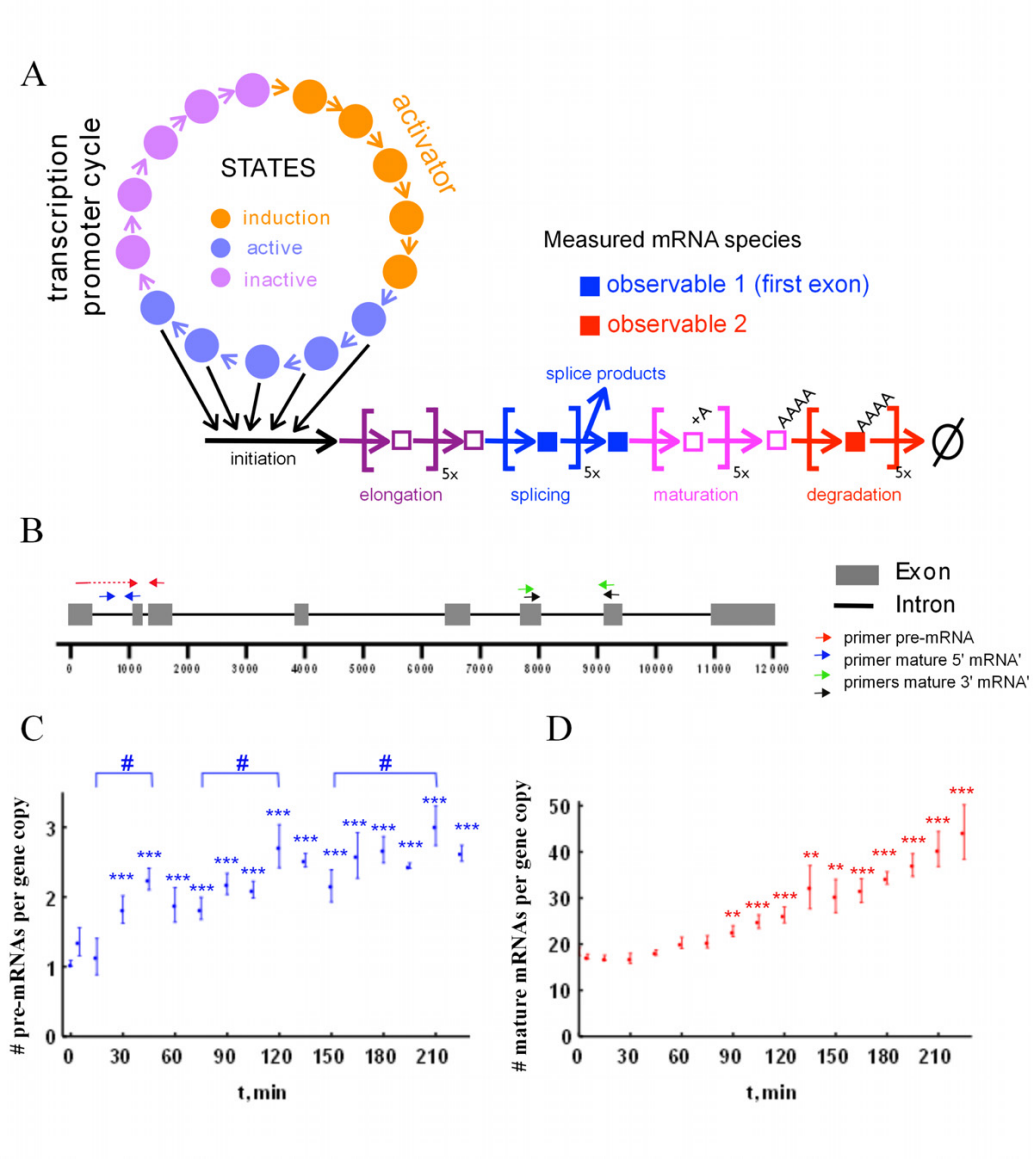
A mathematical model of transcriptional dynamics of the *ADRP* gene in relation to experimental data on pre-mRNA and mature mRNA accumulation. (**A**) Network diagram of our mathematical model of mRNA dynamics. More details can be found in the text and in the Supplementary Material. (**B**) Schematic outline of the human *ADRP* gene and the primer pairs used for this study. The transcriptional dynamics of pre-mRNA (**C**) or mature *ADRP* mRNA (**D**) was measured in human HepG2 cells, which were stimulated for indicated time points with 100 nM of the PPARδ ligand GW501516. Data points represent the means of at least six biological replicates, which were corrected for outliers; error bars represent the standard error of the mean. Two-tailed, paired Student's *t*-tests were performed to determine the significance of the ligand-dependent regulation of the *ADRP* RNA in reference to vehicle (*) and in comparison of peaks to the minima (#): *(#) *P* < 0.05, **(##) *P* < 0.01, ***(###) *P* < 0.001.

We proposed a model for mRNA dynamics, which consists of a 15-state transcription promoter cycle (Figure 1A) to account for possible multiple chromatin states of the promoter. The five states of the ‘induction phase’ (marked orange) have transition rates that depend on the presence of transcription activators. The subsequent five states of the ‘activation phase’ (marked blue) contribute equally to transcription initiation. The subsequent elongation, splicing, maturation and degradation are all described by first order steps in a sequence, which captures in a simple fashion the stepwise nature of these processes and can account for the delays in the experimental data. This means that in this model, mRNA species are observed with a delay with respect to gene activation time. In the third ‘inactivation phase’ (marked violet) the regulatory regions of the gene undergo changes independent of the transcription factor activity. To allow for parameter estimation from experimental data, the model should not contain too many parameters to prevent over fitting and also not too few to make sure it can capture the main features of mRNA dynamics. In the Supplemental Material, we outline how we decided on the final mathematical model using a model selection and parameter fitting procedure. The mathematical model is based on ordinary differential equations (ODEs) and mass action kinetics. The set of ODEs describing the model is presented in Table 1.

**Table 1.** ODE description of the irreversible multi-step model of mRNA dynamics

Process	Equations
Activation	$\frac{{dx_1^{{\rm act}} }}{{dt}} = k_{{\rm rev}} x_5^{{\rm rev}} (t) - k_{{\rm act}} x_1^{{\rm act}} (t)$
	for *i* = 2:5
	$\frac{{dx_i^{{\rm act}} }}{{dt}} = k_{{\rm act}} x_{i - 1}^{{\rm act}} (t) - k_{{\rm act}} x_i^{{\rm act}} (t)$
Deactivation	$\frac{{dx_1^{{\rm dea}} }}{{dt}} = k_{{\rm act}} x_5^{{\rm act}} (t) - k_{{\rm dea}} x_1^{{\rm dea}} (t)$
	for *i* = 2:5
	$\frac{{dx_i^{{\rm dea}} }}{{dt}} = k_{{\rm dea}} x_{i - 1}^{{\rm dea}} (t) - k_{{\rm dea}} x_i^{{\rm dea}} (t)$
Reversion	$\frac{{dx_1^{{\rm rev}} }}{{dt}} = k_{{\rm dea}} x_5^{{\rm dea}} (t) - k_{{\rm rev}} x_1^{{\rm rev}} (t)$
	for *i* = 2:5
	$\frac{{dx_i^{{\rm rev}} }}{{dt}} = k_{{\rm rev}} x_{i - 1}^{{\rm rev}} (t) - k_{{\rm rev}} x_i^{{\rm rev}} (t)$
Initiation	$\frac{{dx_1^{{\rm elo}} }}{{dt}} = k_{{\rm ini}} \sum\limits_{i = 1}^5 {x_i^{{\rm dea}} (t)} - k_{{\rm elo}} x_1^{{\rm elo}} (t)$
Elongation	for *i* = 2:5
	$\frac{{dx_i^{{\rm elo}} }}{{dt}} = k_{{\rm elo}} x_{i - 1}^{{\rm elo}} (t) - k_{{\rm elo}} x_i^{{\rm elo}} (t)$
Splicing	$\frac{{dx_1^{{\rm spl}} }}{{dt}} = k_{{\rm elo}} x_5^{{\rm elo}} (t) - k_{{\rm spl}} x_1^{{\rm spl}} (t)$
	for *i* = 2:5
	$\frac{{dx_i^{{\rm spl}} }}{{dt}} = k_{{\rm spl}} x_{i - 1}^{{\rm spl}} (t) - k_{{\rm spl}} x_i^{{\rm spl}} (t)$
Maturation	$\frac{{dx_1^{{\rm pro}} }}{{dt}} = k_{{\rm spl}} x_5^{{\rm spl}} (t) - k_{{\rm mat}} x_1^{{\rm pro}} (t)$
	for *i* = 2:5
	$\frac{{dx_i^{{\rm pro}} }}{{dt}} = k_{{\rm mat}} x_{i - 1}^{{\rm pro}} (t) - k_{{\rm mat}} x_i^{{\rm pro}} (t)$
Degradation	$\frac{{dx_1^{{\rm mat}} }}{{dt}} = k_{{\rm mat}} x_5^{{\rm pro}} (t) - k_{\deg } x_1^{{\rm mar}} (t)$
	for *i* = 2:5
	$\frac{{dx_i^{{\rm mat}} }}{{dt}} = k_{\deg } x_{i - 1}^{{\rm mat}} (t) - k_{\deg } x_i^{{\rm mat}} (t)$

The equations describe the model presented in Figure 1A. The activation process corresponds to the induction phase, the deactivation process to the active phase and the reversion process to the inactive phase of the promoter cycle.

**Figure 2.**
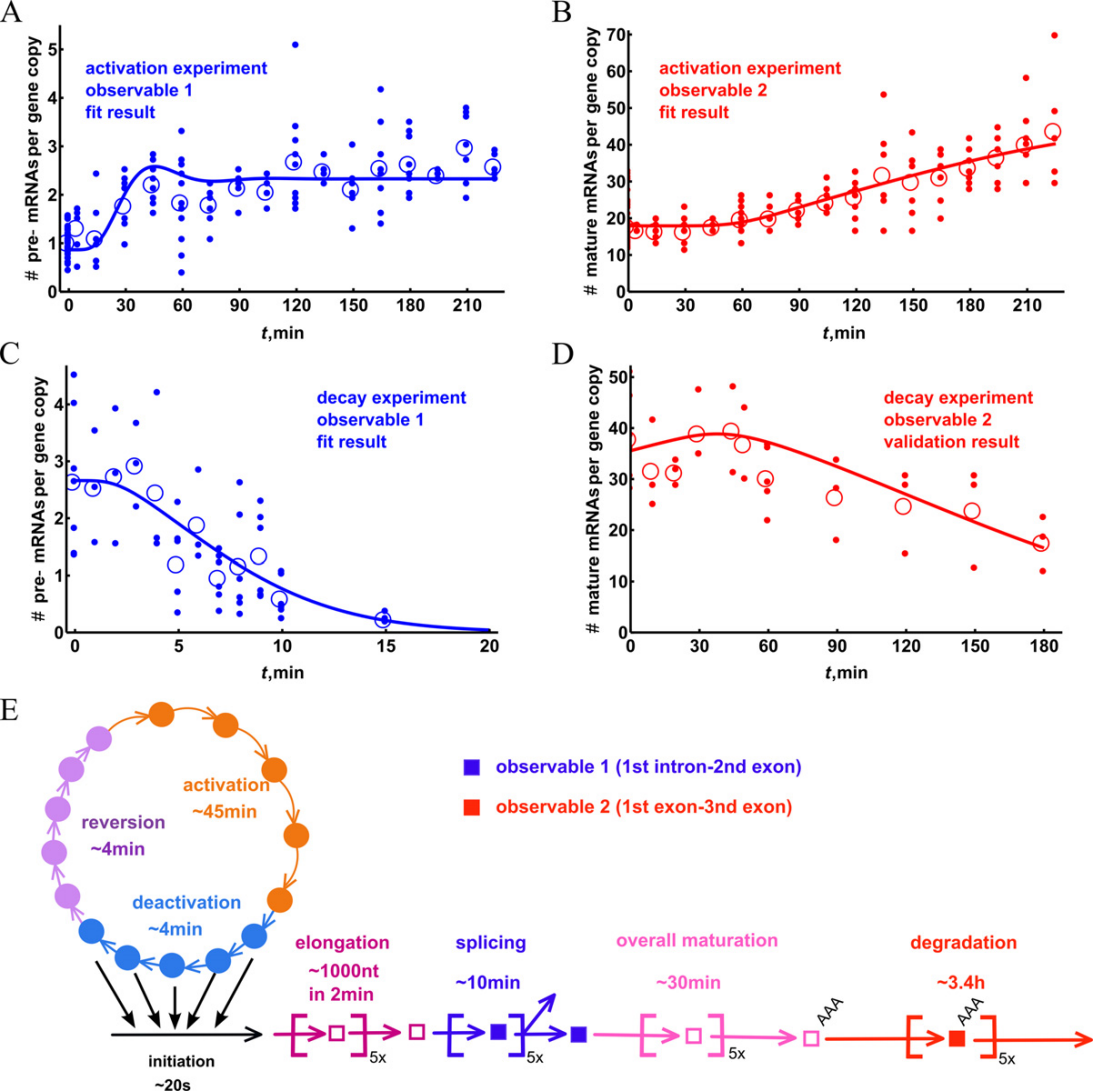
Overview of the experimental data and performance of the mathematical model. For an improved comparison of experimental data and modeling results, the qPCR results for the RNA species were converted to copy numbers per cell. The induction of *ADRP* pre-mRNA (**A**) and mature RNA (**B**) in response to PPARδ ligand treatment are based on the data shown in Figure 1C and D. For comparison, the transcript decay dynamics of *ADRP* pre-mRNA (**C**) and mature RNA (**D**) is displayed. In this experimental series HepG2 cells were first stimulated for 3 h with PPARδ ligand and then treated for indicated time points with the RNA polymerase elongation inhibitor DRB. For each time point the small filled dots indicate individual data points and the circles represent their average. The lines visualize the performance of the fitted model. The curves shown represent the mathematical model in its *n* = 5 version (see Table 1 and Supplemental Material), which was fitted with the data sets A–C and validated with the data for the decay of mature mRNA (D). All the individual data points are reported in Supplementary Tables S3–S5. Please note that the copy numbers of the pre-mRNA decay data (D) were multiplied by 0.4, in order to reconcile the difference with ligand induction experiments (see Supplemental Material Data: conversion error section). The overview of the timescales of different processes in mRNA metabolism that were determined by using parameter estimation procedure is shown in (**E**).

As an experimental system to assess mRNA dynamics we have chosen the human *ADRP* gene (Figure 2B), since it can be largely controlled by a synthetic ligand of its main transcription factor, the nuclear receptor PPARδ. Using qPCR with carefully chosen primer pairs (Supplementary Figure S3A), we performed a first time course measurement in HepG2 cells over 5 h (with 1 h intervals) after ligand addition of synthetic PPARδ ligand GW501516. The data showed that the expression of both the *ADRP* pre-mRNA (Supplementary Figure S3B), i.e. the RNA species before splicing of the first intron (observable 1), and of mature mRNA (Supplementary Figure S3C), i.e. the polyadenylated RNA species after splicing (observable 2), was increased 2- to 3-fold. In our preliminary modeling (Supplementary Figures S4 and Table S2), we found that, in order to discriminate between various models, we would need a higher time resolution. Therefore, we repeated the time course for qPCR quantification of the expression of *ADRP* pre-mRNA (Figure 1C) and of mature mRNA (Figure 1D) with 15 min intervals, but focused on the first 225 minutes after the perturbation of HepG2 cells with PPARδ ligand.

We observed that the deviation between individual experimental results was relatively high (average coefficient of variation was 20–25%, but also up to 40% for some time points), especially for pre-mRNA, which is less abundant. A possible explanation for this would be quite complex dynamics of the system that varies in its timing between individual biological-replicate experiments. Because our modeling showed that such damped oscillatory dynamics could occur in our kinetic models (see below), we decided not to discard the data as ‘too noisy’, but to analyze them in further detail. The first significant induction of the pre-mRNA was observed at 30 min (Figure 1C), which fits the preliminary data of Supplementary Figure S3B. However, the four times more detailed time course demonstrated that the overall increase did not seem to be monotonous. The peaks at 45, 120 and 210 min were found to be significantly different in comparison to the minima at 15, 75 and 150 min, respectively. This is in line with other studies showing dynamic accumulation of RNA transcripts (10). In contrast, mature RNA (Figure 1D) increased only gradually, but the first significant increase was detected only at time point 90 min, i.e. with a 60-min delay compared to the pre-mRNA. This occurrence of a delay in mRNA dynamics necessitates a model with reaction sequences.

In order to make the qPCR results amenable to mathematical modeling, we expressed the abundance of the RNA species in terms of copy numbers per cell. First, we estimated the number of RNA molecules in untreated cells from the standard curve of the qPCR values versus the copy number (the full description of the procedure is shown in the Supplemental Material). Then we used the fold induction by ligand treatment to calculate the copy number for the ligand-treated samples. We found the average number of mature RNAs per cell to be around 18 (Figure 2B), which is approximately 15 times higher than the number of pre-mRNA molecules (on average 1.1 copies per cell, Figure 2A). The experimental limitations led to a rather high error of the copy number determinations (Supplementary Figure S5), but the lowest estimate of the copy number mature mRNAs was still 5-times higher than the highest of the pre-mRNA.

To further enlarge the experimental dataset on transcriptional dynamics, we measured the decay of mRNA species adding the RNA polymerase II elongation inhibitor DRB (31) after HepG2 cells had been treated for 3 h with PPARδ ligand. This inhibitor has been shown to affect several regulatory factors of the RNA polymerases (31,32). The decay kinetics of the *ADRP* RNA species were measured by qPCR relative to those of the housekeeping gene *RPLP0* (Figure 2C and D). The degradation of the known highly stable gene glyceraldehyde-3-phosphate dehydrogenase (*GAPDH*) was measured in relation to *RPLP0* as well; the level of *GAPDH* mRNA expression did not change during 5 h of DRB treatment (Supplementary Figure S6), confirming stability of the housekeeping gene. We observed a relatively fast decay of *ADRP* pre-mRNA on a scale of 10 min after a delay of a few minutes (Figure 2C). We attribute this delay to the production of pre-mRNA by RNA polymerases that already have escaped transcription start site (TSS) of the *ADRP* gene, but have not yet reached the second exon. For mature *ADRP* mRNA we observed a delay of 1 h prior to the start of the degradation, which showed a far slower kinetics than that of the pre-mRNA (Figure 2D).

### Parameter estimation of the mathematical model

Seven mathematical models, one simple model with single step transcription initiation (designated *n* = 0) based on the scheme shown in Supplementary Figure S4A, one slightly more complex model that includes two-step initiation (designated *n* = 2, based on a scheme in Supplementary Figure S4B) and five multi-step models that are based on the design outlined in Figure 1A and Supplementary Figure S4C, were fitted to the experimental data shown in Figure 2A–C. The multi-step models differed in the number of sub-states (*n* = 1, 3, 5, 10 or 20) that they were allowed to have in each of the processes (promoter activation, splicing, elongation, etc.). The model *n* = 0 had only two states and corresponded to a TSS region being constantly active, while the *n* = 2 model had a total of five states and included two steps activation of the promoter. For each of these models, the kinetic parameters as well as some initial conditions of the mathematical model were estimated (Supplementary Table S6) by minimizing a weighted least squares sum that quantifies the distance between model output and experimental data. We used a global minimization algorithm called Controlled Random Search (CRS) (33), which was modified to enhance performance (see Supplemental Material, Supplementary Figure S7). We compared different models on the basis of their minimized weighted sum of the squared differences between the best fit and the experimental data points, their runs-tests and the values of Akaike Information Criterion (AIC) (shown in Supplementary Table S7). The AIC corrects for the complexity differences of the models. We found that based on these criteria, the simple models, i.e. with *n* = 0 and *n* = 2 steps, described the data less well than the models with more steps. The multi-step models, however, do not produce very different values for any of our statistical measures, although the *n* = 10 and *n* = 20 models did indicate pronounced oscillatory dynamics (Supplementary Figure S8). This suggests that transcriptional dynamics is reliant on a multi-state sequential process. Based on the combination of scores for the residual, runs-test, AIC and on visual inspection (spurious oscillations) we decided to focus on the *n* = 5 model. In this model (see Figure 1A), all states have five sub-states and the activation by the ligand directly changes the rate of the first two transitions in the promoter activation process.

We next analyzed the parameter fits to the experimental data. We found optimal parameter sets that minimize the objective function, which is a measure for the distance between model and the data. Accordingly, the optimal parameter set that best describes the data leads to a minimum of the objective function. If this function depends strongly on a specific parameter value, then this parameter value is strongly constrained by the data and otherwise not. An assessment of (*a posteriori*) parameter identifiability, which evaluates the dependency of the objective function on the parameter values, indicates that not all parameter values could be estimated equally well from the experimental data. One of the ways to carry out an *a posteriori* parameter identifiability analysis is to compute the independent and dependent confidence intervals for the parameters (17). The calculations for the *n* = 5 model indicate that certain parameter values (*k*_elo_, *k*_spl_, *k*_mat_ and *k*_deg_) are fairly well constrained as both their independent and dependent confidence intervals are similar and relatively small (Table 2). By contrast, *k*_rev_, *k*_dea_ and *k*_ini_, were poorly constrained and their independent confidence intervals are much bigger than the dependent intervals, which indicates a strong co-dependence between these parameters with others (also reflected by the covariance coefficients, Supplementary Table S8). The exact value of *k*_act_ kinetic parameter proved to be co-dependent as well, in particular on other promoter constants, but less strongly. In Table 2, we compare the fitted parameters to their values reported in the literature and find that the estimated values lie within ranges measured experimentally. This is true both for the parameters that are well constrained and those that are poorly constrained by the fitting. This indicates that the fitting procedure and the model that we chose sketches a realistic picture of the underlying mechanistic biology. The graphic representation of the timescales of various processes in mRNA metabolism is given in Figure 2E.

**Table 2.** Overview of the fitted model parameters and comparison to values reported in the literature

Process	Activation	Deactivation	Reversion	Initiation	Elongation	Splicing	Maturation	Degradation
Value of fitted rate constant, min^−1^	0.106	1.3	1.145	3.436	2.272 0.45 kb/min^b^	0.512	0.161	0.025
Dependent confidence interval	0.0022	0.0256	0.2840	0.0627	0.4780	0.0158	0.0337	0.0008
Independent confidence interval	0.0499	6.8337	4.9264	18.9385	0.7861	0.0817	0.0940	0.0047
Characteristic time, min^a^	47.1	3.8	4	0.27	2.2	9.8	31.1	202.4
Reported range for characteristic time	10–150 min (38)	1–15 min (38)	N/A	0.8 min (41)	0.38 -3 kb/min (40,41)	5–10 min (40)	–	30–900 min (∼300^c^ min) (25,46)

^a^The characteristic times are defined as *n*/*k*, with n as the number of elementary steps in the processes (always *n* = 5 except for initiation where *n* = 1).

^b^The elongation rate in kb/min as calculated from the fitted characteristic time (2.2 min) and the length of the amplified fragment (1178 nt).

^c^Degradation rate found specifically for *ADRP* mRNA.

### Validation of the mathematical model

In order to validate the mathematical model, we compared the simulated and experimental profiles of mature mRNA decay after DRB application (Figure 2D). The overall degradation timescale of the mature mRNA was in the order of several hours and displayed a pronounced delay of about 1 h. This supports the hypothesis of the influx of pre-mRNA that is still in the process of elongation/maturation (unaffected by DRB) into the mature mRNA pool. The prediction of mature mRNA decay was very close to the experimentally measured values (Figure 2D) and the sensitivity of the model prediction to parameter perturbation was similar to the prediction to the sensitivity of the model fit (Supplementary Figures S9 and S10). We found the half-life of the mature mRNA to be approximately 3 h, which is considerably shorter than the 5 h reported previously (25). The reason for the discrepancy may lie in differences between the cell lines used, but may also be a result of the degradation rate fitting procedure that does take into account any delay due to maturation. If we fit *ADRP* mature mRNA degradation with a single exponent, we obtained a half-life of ∼4 h, which is closer to the literature value (25).

### RNA degradation mechanism

The degradation of pre-mRNA occurs primarily due to mistakes in processing pre-mRNA, such as lack of capping, aberrant folding, failure of export, etc. (34). We therefore do not expect this degradation to be a significant contributor to the degradation of pre-mRNA and consider only degradation of mature mRNA. The degradation of mature mRNA can proceed either in the 5′ to 3′ direction, starting with de-capping, or in the 3′ to 5′ direction, starting with removal of polyA tail. In order to test which of them is involved in *ADRP* mRNA degradation, we compared the amount of the polyA-containing mRNA detected by either 5′ (exon 1) or 3′ (exon 6) fragments of the *ADRP* gene. If the degradation occurs via 5′ to 3′-pathway we would expect that the amount of exon 6 fragments would be higher, since the degradation of the exon1 would occur first. In case of 5′ to 3′-degradation, the amounts of both fragments are expected to be equal; since, after removal of the polyA tail neither exon can be detected. The average amounts of the mature mRNA species detected, using exons 1, 3 and exons 5, 6 primers, were very close (18 and 25 copies/cell, respectively) and could not be distinguished significantly, thus strongly suggesting that 3′ to 5′ degradation mechanism occurs (Supplementary Figure S5). Additional evidence is provided by the lack of significant difference in the accumulation dynamics of the exon 1 and exon 6 containing mature mRNA. (Supplementary Figure S11B), which is in line with the 5′ to 3′ degradation mechanism. As a control, to confirm that the positioning of the 3′-primers does not influence the result of the qPCR, we designed another primer pair in close proximity to the initial set (Supplementary Figure S11A), but did not observe a significantly different result (Supplementary Figure S11C).

Furthermore, we tried to determine the amount of the mRNA species that is partially degraded form its 3′ end, which would allow estimating the degradation rate of mRNA after the removal of polyA tail. Because all mRNA species are produced at the same rate by the initiation process, the difference between their steady state levels is proportionate to difference in their decay rates. The copy number of partially degraded species can be calculated by taking the difference between the measured exons 5, 6 copy number in the total mRNA fraction and in the polyadenylated fraction at *t* = 0. This number also includes unspliced mRNA, but we expect it to be very low, in the range of few molecules based on the measured values (Supplementary Figure S5). Surprisingly, the estimated copy number (21) was comparable to that of polyadenylated mRNA (18) (Supplementary Figure S5), which suggests that the steady-state degradation rate of mRNA, after removal of the polyA tail, is similar to that of the removal of polyA tail. This finding is surprising, as one would expect an mRNA that is no longer used for the translation to be degraded quickly.

### Using the mathematical model to detect the site of perturbation

**Figure 3.**
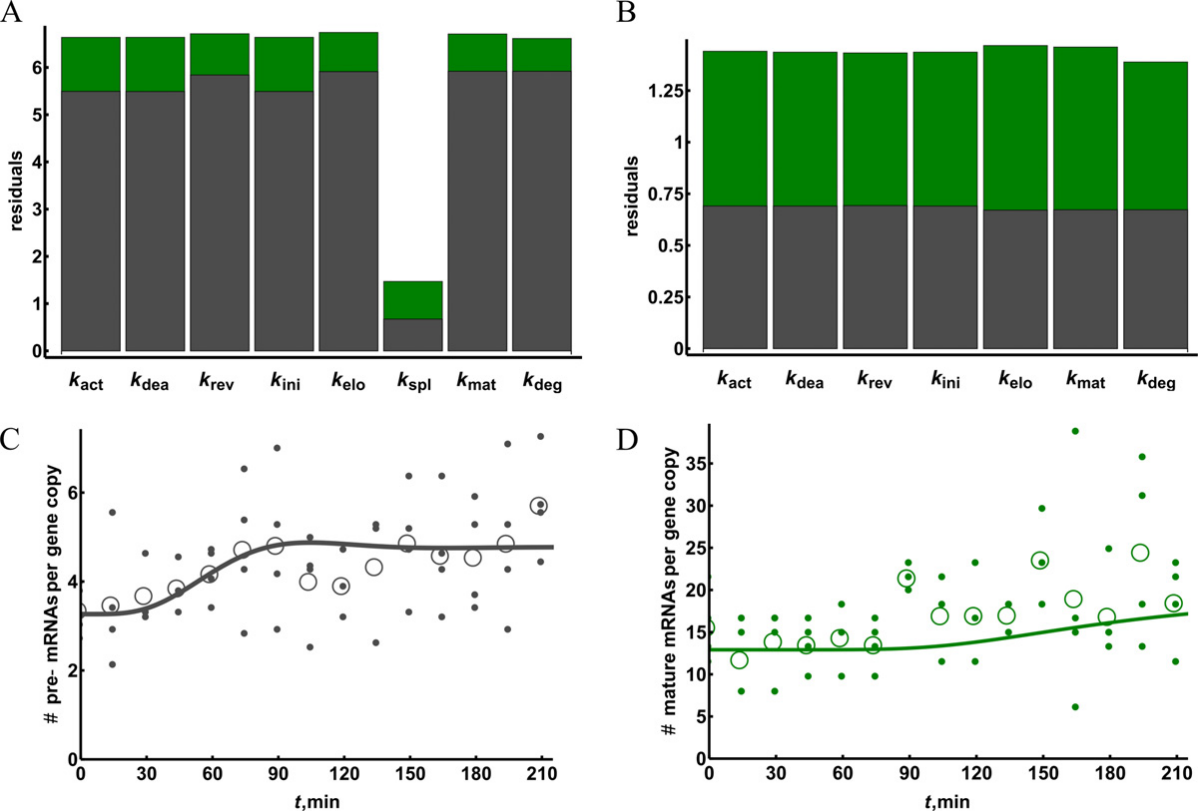
Re-fitting the model can indicate the perturbation in the system. Human HepG2 cells were pre-incubated with 100 μM isoginkgetin for 16 h and subsequently treated with PPARδ ligand for the indicated time periods. qPCR was performed in order to measure pre-mRNA (**A**) and mature mRNA levels (**B**) and copy numbers were calculated. Small filled circles represent all data points from at least four biological experiments corrected for outlier and empty circles indicate their mean. All the individual data points are reported in Supplementary Tables S9. The mathematical model (adjusted for apparent fold induction) was fitted to the data by changing a single constant at a time and the quality of the fit was compared for pre-mRNA (grey) and mature mRNA (green) time courses (**C**). The best model obtained by lowering the splicing rate constant was further tested for fit improvement by changing the remaining constants (**D**). The best fit given by adjusting both splicing and degradation constants is also shown for the pre-mRNA (A) and mature mRNA (B) time courses.

One application of mathematical modeling and parameter estimation that we foresee is the identification of the site of perturbation from comparisons of unperturbed and perturbed mRNA dynamics. To test whether the mathematical model that we developed can be used for this purpose, we measured the transcriptional dynamics upon gene activation in the presence of a splicing inhibitor. We used the splicing inhibitor isoginkgetin, which targets both the major and minor spliceosomes (35), but we will here pretend to be ignorant of this information and then see if the model can detect the perturbation in the experimental data. We found that short inhibitor treatment (3 h) and co-treatment with PPARδ ligand were insufficient to affect pre-mRNA and mature RNA levels (Supplementary Figure S12). Therefore, we repeated the experiment with a pre-treatment of 16 h (Figure 3). Under this condition, we observed 3-fold increased pre-RNA levels (Figure 3A), while the level of mature mRNA decreased slightly below that of the non-treated control (Figure 3B). The subsequent treatment with the PPARδ ligand caused an increase of the pre-mRNA with a broad peak observed at 75–105 min (Figure 3A). The overall induction of mature mRNA was lower than in the absence of isoginkgetin, possibly due to non-specific effects of the inhibitor.

We then re-fitted the best fitting model (i.e. the one shown in Figure 2) by changing a single parameter at a time (curves in Figure 3A and B). We compared the resulting quality of fit for pre-mRNA and mature mRNA: the only parameter that upon adjustment gave a considerably better fit of the model to the pre-mRNA as well as a better overall fit was *k*_spl_, unambiguously pointing to the perturbation of splicing as the action of the added inhibitor (Figure 3C). The resulting splicing constant was around four times lower than that in the fit of the data in the absence of the added inhibitor, suggesting that the added inhibitor concentration was sufficient to inhibit splicing by some 75%. We further tested if the fit could be improved further by changing other constants in addition to *k*_spl_ (Figure 3D). Only a slight improvement could be achieved, and only by increasing *k*_deg_, but this was below the level of significance (Supplementary Table S10). The overall goodness-of-fit is worse for the splicing inhibition data when compared to ligand induction time course data. This is likely due to higher noise in the data as the average coefficient of variation for the mature mRNA time course in the splicing experiment is 0.3 compared to 0.2 in the ligand induction experiment. We therefore conclude that the model successfully predicts the primary site of the applied perturbation, and that it is robust against spurious ‘prediction’ of other sites.

Next, we analyzed whether the prediction depended on the particular set of parameters by changing each parameter independently and comparing the effect on the quality of the original model fit and on that of the adjusted model. With the independent parameter perturbations the decrease in the original fit quality caused a proportional decrease in the adjusted fit (Supplementary Figure S13), as the sensitivity profiles of the original and refitted models are very similar (Supplementary Figures S9 and S14). This was not the case, when the similar procedure was used to estimate the occurrence of correlated changes of the co-dependent parameters (*k*_act_*, k*_dea_*, k*_ini_ or *k*_rev_). If the co-dependent constants were changed so that the overall rate of mRNA production stayed the same, the relative decrease in adjusted model fit quality was much lower (Supplementary Figure S14). This means that worse fitting models could be successfully fitted to the data from the splicing inhibition experiment. The latter could have been expected due to poor constraints of promoter constants when changed in a co-dependent manner. This indicates that if the site of perturbation was at the promoter level, it would likely be impossible to distinguish between changes in, for example, initiation and promoter deactivation rates.

## DISCUSSION

In this paper, we integrated a mathematical modeling and parameter estimation procedure with targeted experiments in order to understand the dynamics of a nascent mRNA population upon activation of transcription. The model is capable of capturing the observed experimental delays in the appearance of mature mRNA by explicitly incorporating the essential processes of mRNA transcription and processing. The transcriptional dynamics of the primary PPARδ target gene, *ADRP*, in HepG2 human liver cells served as experimental model. By comparing the mRNA dynamics of perturbed and non-perturbed cells, our mathematical model correctly predicted the site of perturbation.

The quite noisy experimental data provided a challenge. Under normal conditions, the noisiness of the data would have precluded us from drawing any conclusion. However, by implementing mathematical models we were able to reach two important conclusions, without being disturbed by the noise: the transcriptional dynamics reflects a sequential process of more than one step (the *n* = 0 had a much worse fit than other models) and the applied inhibitor could be identified as acting on splicing. We were able to make these conclusions because fits of the mathematical models can be evaluated and compared in objective ways that are free from preconceptions. This means that modeling enables extraction of information even from noisy data and correctly identifies the perturbations applied to the system.

The transcription parameters estimated by fitting the model to the experimental data come close to values reported in the literature. We estimated the promoter cycling time to be around 60 to 70 min (calculated as the sum of three phases of the promoter cycle). This is in agreement with the timescales indicated by the chromatin immunoprecipitation experiments that measure presence of transcription factors on their regulatory binding sites. Several studies found periodic binding of various specific and general transcription factors in the range of 45–90 min (11,36,37). A study in yeast also described oscillation in the mRNA of a copper-regulated gene with a period of 50 min, which is produced by the synchronous bursts of mRNA production in cells (10). Although in our experimental system there is evidence for the multi-step promoter cycle, we could not observe significant oscillations of mature mRNA. This could result from relatively irreproducible promoter cycle times or due to too much noise in the data. Recently, time resolved transcriptional activity at a single cell level has been measured for several genes (38), which showed promoter cycle times within the range found in this study. This study also found that the promoter switching times were non-exponentially distributed further suggesting a multi-step mechanism is involved. The initiation time of 0.8 min on a eukaryotic promoter, based on the *in vivo* single cell measurements, is also reasonably close to our estimates. As has been shown previously (39), it is not possible to establish the mRNA burst size accurately from the steady-state mRNA distributions. Therefore the found range of 5–40 molecules is only suggestive but fits well with the calculated (a ratio of characteristic times of the promoter deactivation and initiation) burst size of 12 mRNA molecules.

The estimated RNA polymerase II elongation rate in our system was 0.45 kb/min, which is considerably slower than what has been reported for mammalian system, ranging from 1 to 4 kb/min (40). However, although the actual elongation rate of a mammalian RNA polymerase II is 4 kb/min, due to pausing the average progression rate is only 0.4 kb/min (41), i.e. close to our estimation. With this progression rate it would take RNA polymerase II about 40 min to complete the transcription of the *ADRP* gene, which is less than the observed maturation time of 60 min. This discrepancy might be due to a higher frequency of pausing, to additional time spent on 3′ processing or on post-transcriptional splicing of some of the introns. The splicing of the first intron occurs co-transcriptionally as the measured excision rate (∼8 min) is much faster than the overall maturation time. Recent large scale study has provided support to the hypothesis that most of the splicing indeed occurs co-transcriptionally (42). The splicing rate that we found is within the range of 5–10 min described for various introns in several human genes (40).

Our results showed that it is possible to estimate all main parameters of the transcription process from a modest data set albeit with variable accuracy. We also observed that an RNA polymerase II elongation inhibitor can be used for simultaneous measurement of the polymerase progression (due to delays caused by elongation), splicing and mRNA degradation rates. Delays in the mRNA decay data can affect the measured degradation rates if not taken into the account; a number of studies indeed show such delays in mature mRNA decay profiles (43–45). We believe that our approach may well be useful for simultaneous measurement of various mRNA metabolism properties of many genes. Another important application provided by modelling could be identification of the source of perturbation in disease or drug treatment using large dynamic mRNA datasets.

## SUPPLEMENTARY DATA

Supplementary Data are available at NAR Online.

SUPPLEMENTARY DATA
