# Supplementary material for: Tracing the molecular basis of transcriptional dynamics in noisy data by using an experiment-based mathematical model
Source: Nucleic Acids Res. 2014 Dec 3;43(1):153–61. doi: 10.1093/nar/gku1272 (PMC4288170; doi:10.1093/nar/gku1272)
Supplement: SUPPLEMENTARY DATA [file supp_43_1_153__index.html]

Tracing the molecular basis of transcriptional dynamics in noisy data by using an experiment-based mathematical model — Tracing the molecular basis of transcriptional dynamics in noisy data by using an experiment-based mathematical model — Tracing the molecular basis of transcriptional dynamics in noisy data by using an experiment-based mathematical model — SUPPLEMENTARY DATA 

# Tracing the molecular basis of transcriptional dynamics in noisy data by using an experiment-based mathematical model

## SUPPLEMENTARY DATA

**Files in this Data Supplement:**

- SUPPLEMENTARY DATA
- SUPPLEMENTARY DATA
