# Supplementary material for: Tracing the molecular basis of transcriptional dynamics in noisy data by using an experiment-based mathematical model
Source: Nucleic Acids Res. 2014 Dec 3;43(1):153–61. doi: 10.1093/nar/gku1272 (PMC4288170; doi:10.1093/nar/gku1272)
Supplement: SUPPLEMENTARY DATA [file supp_gku1272_nar-03211-n-2014-File005.pdf]

Supplemental Material

Comparing model structures

To illustrate the importance of model choice, we compare two alternative models of mRNA dynamics. One model is highly simplified and considers continuous ('constitutive') mRNA production, first-order maturation and first-order degradation (Fig. S4A). It is described by the following equations:

$$\frac{dx^{pro}}{dt} = k_{ini} - k_{mat}x^{pro}(t) \quad (1a)$$

$$\frac{dx^{mat}}{dt} = k_{mat}x^{pro}(t) - k_{deg}x^{mat}(t) \quad (1b)$$

Fig. S4 shows the pre-mRNA species (x^{pro}, G-I, dotted) and mature mRNA (x^{mat}, D-F, dotted).

The second model takes into account a multi-step promoter cycle, mRNA maturation and degradation (Fig. S4C). Here we explore a general multi-step model, which we will make more detailed (including explicitly elongation and splicing) in the subsequent sections for the purposes of data fitting. The system is described by the following set of equations:

$$\frac{dx_1^{act}}{dt} = k_{dea}x_5^{dea}(t) - k_{act}x_1^{act}(t) \quad (2a)$$

for i=2:5

$$\frac{dx_i^{act}}{dt} = k_{act}x_{i-1}^{act}(t) - k_{act}x_i^{act}(t)$$

$$\frac{dx_1^{dea}}{dt} = k_{act}x_5^{act}(t) - k_{dea}x_1^{dea}(t) \quad (2b)$$

for i=2:5

$$\frac{dx_i^{dea}}{dt} = k_{dea}x_{i-1}^{dea}(t) - k_{dea}x_i^{dea}(t)$$

$$\frac{dx_1^{pro}}{dt} = k_{ini}\sum_{i=1}^{5}x_i^{dea}(t) - k_{mat}x_1^{pro}(t) \quad (2c)$$

for i=2:15

$$\frac{dx_i^{pro}}{dt} = k_{mat}x_{i-1}^{pro}(t) - k_{mat}x_i^{pro}(t)$$

$$\frac{dx_1^{mat}}{dt} = k_{mat}x_{15}^{pro}(t) - k_{\deg}x_1^{mat}(t) \quad (2d)$$

for i=2:15

$$\frac{dx_i^{mat}}{dt} = k_{\deg}x_{i-1}^{mat}(t) - k_{\deg}x_i^{\deg}(t)$$

The pre-mRNA species plotted in Figs. S4G-I (solid) is defined as $\sum_{i=1}^{15} x^{pro}$ and mature mRNA (Fig. S4D-F, solid) as $\sum_{i=1}^{15} x^{mat}$. Parameters used for the simulation of the various time courses are displayed in Table S2. At the start of the simulation all the variables are 0 except for $i = 1$: $x_i^{dea} = 0.2$.

In Figs. S4D-I the time courses of the pre-mRNA and mature mRNA abundance are shown upon activation. The rate constants were adjusted so that the overall production, maturation and degradation rates and starting/ending steady state values of the mature mRNA are the same. Notably, for the multi-step model the dynamics vary significantly depending on the parameter regime, i.e. the relative rates of promoter cycling, mRNA maturation and degradation, but are always distinct from those of the simplified single step model. If degradation and maturation occur fast compared to the promoter cycling, both the mRNA and the pre-mRNA accumulation displays damped oscillatory dynamics for the more complicated model (Figs. S4D and G). This is explained by the fact that simultaneously activated cells go through a round of mRNA production leading to a rise and then during promoter silent periods part of the pre-mRNA is spliced and the mature mRNA is degraded resulting in a decrease (Fig. S4D). When the promoter is active again, it leads to an increase of RNA production and so on. In the single step model, on the other hand, the parameter regimes only affect the accumulation rate but not the overall form of the profile; in all cases both the pre-mRNA and mature mRNA increase monotonously without damped oscillations (Figs. S4D-I).

As shown above, a number of our ODE models produce damped oscillations in the dynamics of transcript concentrations. We emphasize that those models describe the average behaviour of a population of cells. Hence, it is relevant to shortly discuss some of the conditions that the single cells would have to obey in order for a population of them to show (damped) oscillatory dynamics of the average transcript concentrations. Firstly, the initial conditions of the cells should be close to identical when transcription is activated. Otherwise single cells would display different dynamics from the onset. Secondly, the cells induced at the same time and from similar state can stay in synchrony if the waiting times of the sequential processes that they carry out are 'precise'. By precise we mean that the cells have similar waiting times for the same processes, such as promoter activation, transcript elongation, etc. This is not to be expected for first order reactions, for which the standard deviation in the waiting time equals the mean time; so it has a coefficient of variation (CV) of 1. Thus single reactions are extremely noisy and do not lead to synchrony between cells. However, when several (for instance, N) of those reactions are occurring in a sequence, then the waiting time of the complete sequence becomes much more precise: it has a coefficient of variation of 1/N. Therefore, multistep processes that occur in transcription initiation cycle and elongation can lead to passive, transient synchronisation of cells. Finally, due to small stochastic differences between cells – that inevitable will occur and accumulate – in all cases, the oscillations at the level of single cells will slowly desynchronize, which leads to dampening on the population levels.

Data analysis

We used data from two types of time course experiments: ligand activation and transcription inhibition (degradation). From both experimental series at each time point the Ct values of pre-mRNA and mature mRNA of the human *ADRP* gene are available as well as the Ct-values of housekeeping control genes. The Ct-values are converted to copy numbers as demonstrated below.

Let C_M be the Ct value of the RNA under investigation, C_H the Ct of the RNA of the housekeeping gene (which does not change expression during the experiment, see Fig. S1) and $M = C_M - C_H$. We define the expression level 'E' as $E = 2^{-M}$, which is the expression level relative to that of the housekeeping gene. This procedure corrects for the technical variation due to sample preparation. For each data point at t > 0 we acquire an M (and a corresponding E) value of the experimental (ligand or inhibitor-treated) sample. This is then corrected by the average M of the control samples ($\overline{M}_{cntr}$) belonging to the particular repeat of the experiment, giving $dM = M - \overline{M}_{cntr}$. The fold induction R is defined as $R = 2^{-dM}$. This procedure corrects for the biological variation (different cell culture) between the experiments. Only dM (and R) values can be compared between the experiments and not C_M or M values. However, to estimate the copy number from the standard curve, a C_M value is required. Thus the C_M's are calculated back using average M and C_H values of all the control samples: $C'_m = dM - \overline{M}_{cntr} - \overline{C_H}$. In case of t = 0 (or control samples), the calculation is $C'_M = M - \overline{C_H}$. The copy number is provided by:

$$t = 0: a \cdot 10^{\frac{b-(M-\overline{C_H})}{c}}$$

$$t > 0: a \cdot 10^{\frac{b-(M-\overline{M}_{cntr}-\overline{C_H})}{c}}$$

where *a, b*, and *c* are component-dependent experimental constants and the bar indicates the mean value. The *b* (the Ct value, if the copy number equals 1 in the calibration experiment) and *c* (the exponential factor in the PCR, theoretically equal to $-^2\log 10 = -3.32$) values come from calibration experiments that establish the relationship between Ct-values and copy numbers in the cDNA sample (Fig. S2). They allow determining the number for the respective molecule in the sample. Factor *a* is then used to obtain the copy number per cell as it is a product of the multiplication of: (i) the sample dilution factor (2,500 for mature mRNA, 625 for pre-mRNA), (ii) the inverse cDNA synthesis efficiency coefficient (1 for mature and 0.1 for pre-mRNA) and (iii) the inverse of the cell number in the culture (10^6 cells per culture dish). The cDNA synthesis efficiency coefficients are experimentally determined using an *in vitro* synthesized RNA added in known concentration to a cDNA synthesis mixture (see Methods for details). The resulting cDNA copy number was measured using standard curve (Fig. S2D). Measured cDNA amount divided by added amount of RNA gives the cDNA synthesis efficiency factor.

These factors are a = 0.00125, b = 38.70, c = -3.69 for mature mRNA (*b* and *c* are based on

the average fit of the two calibration experiments of Fig. S2B) and a = 0.003125, b = 38.93, c = -3.70 (based on average fit of the calibration experiment in Fig. S2A) for the pre-mRNA time course experiments.

For the ligand activation experiment we have 17 time-points for both pre-mature and mature mRNA, t^l = (0, 5, 15, (30-210), 225); for the degradation experiment we have for the pre-mature mRNA 12 time-points, $t^{d,pR}$ = (0, (1-9), 10, 15) and for the mature mRNA 11 time-points, $t^{d,mR}$ = (0, (10-60) 90, 120, 150, 180). All time-points consist of several data points (2-13), for the initial steady state in the ligand activation experiment there are around 45 data points (see the column 'n' in Tables S3-4).

In our fitting procedure we use the copy number data, see Tables S3, S4 and S5.

We discuss three sources of error in the data.

Device error: The error resulting from the measurement device. For each data point the Ct values measured are an average over 3 measurements. We have neglected this error as it is relatively small (the average standard deviation is 0.13, which would result in a ±0.1 copy number error for pre-mRNA and ±1.5 copy number error for mature mRNA). For comparison, the average standard deviation between replicas of a single time point in the ligand induction time course is 0.52 for pre-mRNA and 5.8 for mature mRNA.

Conversion error: The error resulting from the conversion from Ct-values to copy numbers. The copy number of pre-mRNA at t = 180 min should be equal to the copy number of the starting value in the decay experiment for the pre-mRNA measurements. This was not the case in the primary data. The likely reason for this difference is that due to technical reasons there are several missing *M* values for untreated samples, which are necessary for the *dM* calculation that corrects for biological variation. Instead an average of all the available M values were used for *dM* and subsequent copynumber calculations. In case of mature mRNA degradation, where *M* values for all replicates are available, no difference between t=180 of ligand treatment and t=0 of the decay timecourse is observed. We have corrected for the discrepancy between t=180 of ligand treatment and t=0 of the decay timecourse by multiplying the copy numbers of the pre-mRNA decay data by a factor (0.3998) such that the average of the pre-mRNA ligand data at t = 180 min became equal to the average of the pre-mRNA decay data at t = 0 of that experiment. With this corrected pre-mRNA decay data we fit our model and we have assumed further conversion errors to be negligible.

Sample error: The error resulting from the variation over cells. We assume the error in the copy number to be independent for each measurement and to be normally distributed per time point.

Plots of all time courses are presented in Figs. 2A-C. In view of the substantial noise, the number of data points per time point is too small to use the mean+sd as fit data. 'Averaging' the standard deviation over more time points is questionable, since for some data courses

(mature mRNA ligand and pre-mRNA degradation) the hypothesis of no correlation between time points is clearly rejected (Pearson correlation between time point and standard deviation for the mature mRNA ligand is 0.72 and for pre-mRNA degradation -0.78). Therefore, we use all data points in a Least Square Estimation fitting procedure, which is a standard procedure for the fitting over-determined systems. Assuming a scaled constant standard deviation for all time point error distributions, this results in a MLE. For the scaling we used a factor of 10 for the mature mRNA data as there is approximately one order of magnitude difference between the mean values of pre-mRNA and mature mRNA.

Mathematical models

In the ligand activation experiment the system is assumed to be initially in steady state. Let f be the ratio between the steady state value of pre-mature mRNA species (mature mRNA is less likely to reach steady state values as it has much slower degradation rate) in the steady states after and before the ligand addition. f is experimentally determined from the ratio of the average of 2-3 replicates of 5 late time points (t = 240, 255, 270, 285 and 300 min) and the average of all the data points at t = 0 (Table S3). For pre-mRNA the average at t ≥ 4 h is found to be 2.88 copies per cell, resulting in f of 2.69.

Model n = 0

The simplest model (see Fig. S4A) is given by the following equations:

$$\frac{dx^{pro}(t)}{dt} = u_{ini} \cdot k_{ini} - k_{mat} \cdot x^{pro}(t) \tag{3a}$$

$$\frac{dx^{mat}(t)}{dt} = k_{mat} \cdot x^{pro}(t) - k_{deg} \cdot x^{mat}(t) \tag{3b}$$

For the ligand activation experiment the input variable u_{ini} = 1, for the degradation experiment, where initiation was stopped abruptly by addition of an inhibitor of initiation, u_{ini}= 0.

Solutions

For this simple system a closed solution still gives insight, the pre-mature mRNA is a simple exponential function and the mature mRNA a combination of two exponentials.

$$x^{pro}(t) = \left(x^{pro}(0) - \frac{u_{ini} \cdot k_{ini}}{k_{mat}}\right) e^{-k_{mat} \cdot t} + \frac{u_{ini} \cdot k_{ini}}{k_{mat}} \tag{4a}$$

$$x^{mat}(t) = (x^{mat}(0) - \frac{k_{mat} \cdot x^{mat}(0) - u_{ini} \cdot k_{ini}}{k_{mat} - k_{deg}} - \frac{u_{ini} \cdot k_{ini}}{k_{deg}})e^{-k_{deg} \cdot t} \cdot k_{deg}$$

$$+ \frac{k_{mat} \cdot x^{mat}(0) - u_{ini} \cdot k_{ini}}{k_{mat} - k_{deg}} e^{-k_{mat} \cdot t} + \frac{u_{ini} \cdot k_{ini}}{k_{deg}} \tag{4b}$$

Initial conditions

In the ligand activation experiment the system is assumed to be initially in steady state, but it is the steady-state condition without ligand addition. Since we assume ligand addition to influence only k_{ini} the steady state before ligand addition should correspond to the solution of

(1a) with k_{ini} replaced by k_{ini}/f. For the degradation experiment the initial states are unknown and have to be fitted to the data. So the initial conditions are given by the following equations:

$$x^{pro}(0) = \frac{u_{ini} \cdot k_{ini}/f}{k_{mat}} + (1 - u_{ini})x_0^{pro} \quad (5a)$$

$$x^{mat}(0) = \frac{u_{ini} \cdot k_{mat} \cdot x^{pro}(0)}{k_{deg}} + (1 - u_{ini})\frac{k_{mat}x_0^{pro}}{k_{deg}} \quad (5b)$$

Where x_0^{pro} is an unknown parameter and f is an experimentally determined value of the RNA fold induction ($f = 2.69$).

Model n=2

The model that includes two step promoter activation (see Fig. S4B) is described by the following ODE system:

$$\frac{dx^{tf}(t)}{dt} = k_{tf} \cdot x^{emp}(t) - k_{pol} \cdot x^{tf}(t) \quad (6a)$$

$$\frac{dx^{pol}(t)}{dt} = k_{pol} \cdot x^{tf}(t) - u_{ini} \cdot k_{ini} \cdot x^{pol}(t) \quad (6b)$$

$$\frac{dx^{emp}(t)}{dt} = u_{ini} \cdot k_{ini} \cdot x^{pol}(t) - k_{tf} \cdot x^{emp}(t) \quad (6c)$$

$$\frac{dx^{mat}(t)}{dt} = u_{ini} \cdot k_{ini} \cdot x^{pol}(t) - k_{mat} \cdot x^{mat}(t) \quad (6d)$$

$$\frac{dx^{deg}(t)}{dt} = k_{mat} \cdot x^{mat}(t) - k_{deg} \cdot x^{deg}(t) \quad (6e)$$

Solutions

For the n=2 simple system a closed solution exists but it does not provide useful insights.

Initial conditions

In the ligand activation experiment the initial state of the system is the steady state before ligand addition. The addition of the ligand is assumed to only have an effect TF binding step. The basal level of the activating transcription factor is lower before ligand addition (hence $k_{tf}^{0} < k_{tf}$). To reach an f-times higher mRNA 'production' rate after ligand addition given two step initiation model, the following relationship between promoter constants should be satisfied:

$$\left(\frac{k_{pol}\, k_{tf}}{k_{pol}\, k_{tf} + k_{pol}\, k_{ini} + k_{ini}\, k_{tf}}\right) = f\left(\frac{k_{pol}\, k_{tf0}}{k_{pol}\, k_{tf0} + k_{pol}\, k_{ini} + k_{ini}\, k_{tf0}}\right) \quad (7)$$

As f is an experientially determined factor ($f = 2.69$), this allows us to find the value of the $k_{tf}{}^{0}$:

$$k_{tf^0} = \frac{k_{pol}\, k_{tf}\, k_{ini}}{f(k_{pol}\, k_{tf} + k_{pol}\, k_{ini} + k_{ini}\, k_{tf}) - (k_{pol}\, k_{tf} + k_{tf}\, k_{ini})} \qquad (8)$$

The initial conditions for system (6) are then given by the steady-state values of system (6) with the promoter equations (9a-c) replaced by:

$$\frac{dx^{tf}(t)}{dt} = k_{tf^0} \cdot x^{emp}(t) - k_{pol} \cdot x^{tf}(t) \qquad (9a)$$

$$x^{tf}(t) + x^{pol}(t) + \cdot\, x^{emp}(t) = 1 \qquad (9b)$$

$$\frac{dx^{emp}(t)}{dt} = u_{ini} \cdot k_{ini} \cdot x^{pol}(t) - k_{tf^0} \cdot x^{emp}(t) \qquad (9c)$$

so that the initial conditions represent system before ligand addition. One of the promoter differential equations (6b) is replaced by mass conservation relation otherwise the system is singular.

We have used the following initial conditions for system (6):

$$x^{tf}(0) = u_{ini} x_{ss0}^{tf} \qquad (10a)$$

$$x^{pol}(0) = u_{ini} x_{ss0}^{pol} \qquad (10b)$$

$$x^{emp}(0) = u_{ini} x_{ss0}^{emp} \qquad (10c)$$

$$x^{pro}(0) = u_{ini} x_{i\,ss0}^{pro} + (1 - u_{ini}) x_0^{pro} \qquad (10d)$$

$$x^{deg}(0) = u_{ini} x_{i\,ss0}^{deg} + (1 - u_{ini}) k_{mat} x_0^{pro} / k_{deg} \qquad (10e)$$

where u_{ini} is defined as above, and the value x_0^{spl} is an unknown parameter to be fitted to the data.

Multi-step models n = 1, 3, 5, 10, 20

The multi-step model (see Fig. S4C) differentiates in the promoter cycle processes between activation, deactivation and reversion, and in the maturation between initiation/elongation, splicing, and maturation. It is given by the following equations:

activation

$$\frac{dx_1^{act}}{dt} = k_{rev} x_{n3}^{rev}(t) - k_{act} x_1^{act}(t)$$

for i=2:n1 (11a)

$$\frac{dx_i^{act}}{dt} = k_{act} x_{i-1}^{act}(t) - k_{act} x_i^{act}(t)$$

deactivation

$$\frac{dx_1^{dea}}{dt} = k_{act} x_{n1}^{act}(t) - k_{dea} x_1^{dea}(t)$$

for i=2:n2 (11b)

$$\frac{dx_i^{dea}}{dt} = k_{dea} x_{i-1}^{dea}(t) - k_{dea} x_i^{dea}(t)$$

reversion

$$\frac{dx_1^{rev}}{dt} = k_{dea} x_{n2}^{dea}(t) - k_{rev} x_1^{rev}(t)$$

for i=2:n3 (11c)

$$\frac{dx_i^{rev}}{dt} = k_{rev} x_{i-1}^{rev}(t) - k_{rev} x_i^{rev}(t)$$

initiation

$$\frac{dx_1^{elo}}{dt} = k_{ini} \sum_{i=1}^{n2} x_i^{dea}(t) - k_{elo} x_1^{elo}(t) \qquad (11d)$$

elongation

for i=2:n4

$$\frac{dx_i^{elo}}{dt} = k_{elo} x_{i-1}^{elo}(t) - k_{elo} x_i^{elo}(t) \qquad (11e)$$

splicing

$$\frac{dx_1^{spl}}{dt} = k_{elo} x_{n4}^{elo}(t) - k_{spl} x_1^{spl}(t) \qquad (11f)$$

for i=2:n5

$$\frac{dx_i^{spl}}{dt} = k_{spl}x_{i-1}^{spl}(t) - k_{spl}x_i^{spl}(t)$$

maturation

$$\frac{dx_1^{pro}}{dt} = k_{spl}x_{n5}^{spl}(t) - k_{mat}x_1^{pro}(t)$$

for i=2:n6 (11g)

$$\frac{dx_i^{pro}}{dt} = k_{mat}x_{i-1}^{pro}(t) - k_{mat}x_i^{pro}(t)$$

degradation

$$\frac{dx_1^{mat}}{dt} = k_{mat}x_{n6}^{pro}(t) - k_{\deg}x_1^{mat}(t)$$

for i=2:n7 (11h)

$$\frac{dx_i^{mat}}{dt} = k_{\deg}x_{i-1}^{mat}(t) - k_{\deg}x_i^{mat}(t)$$

Solution

Also this system of equations has a closed solution. Because we do not find it insightful, we omit this and use numerical solutions only.

Initial conditions

In the ligand activation experiment the initial state of the system is the steady state before ligand addition. The addition of the ligand is assumed to only have an effect on the first $n1^0$ steps of the activation process (see Fig. 1). The basal level of the activating transcription factor is lower in these first steps (hence $k_{act}^0 < k_{act}$); if the ligand is added it reaches approximately the same level as the transcription factor for the second part of the activation. To reach an *f*-times higher 'production' from the promoter cycle, the cycle time should be *f* times as fast, giving the following relationship between the activation rate constants before (k_{act}^0) and after (k_{act}) the ligand addition.

$$f\left(\frac{n1}{k_{act}} + \frac{n2}{k_{dea}} + \frac{n3}{k_{rev}}\right) = \left(\frac{n1^0}{k_{act^0}} + \frac{n1-n1^0}{k_{act}} + \frac{n2}{k_{dea}} + \frac{n3}{k_{rev}}\right) \quad (12)$$

The initial conditions for system (11) are then given by the steady-state values of system (11) with the activation equations (11a-b) replaced by

$$\frac{dx_1^{act}}{dt} = k_{rev}x_{n3}^{rev}(t) - k_{act^0}x_1^{act}(t)$$

$i=2...n1^0$ (13a)

$$\frac{dx_i^{act}}{dt} = k_{act^0} x_{i-1}^{act}(t) - k_{act^0} x_i^{act}(t)$$

$$\frac{dx_{n1^0+1}^{act}}{dt} = k_{act^0} x_{n1^0}^{act}(t) - k_{act} x_{n1^0+1}^{act}(t)$$

$$i = n1^0+2 \ldots n1 \tag{13b}$$

$$\frac{dx_i^{act}}{dt} = k_{act} x_{i-1}^{act}(t) - k_{act} x_i^{act}(t)$$

Note that, if $n1 = n1^0$, all corresponding k_{act} in (11b) should also be replaced by k_{act}^0.

The system is singular because of the promoter cycle. Therefore one of the 'internal' equations, e.g. the first one above, is replaced by the mass conservation relation

$$\sum_{i=1}^{n1} x_i^{act} + \sum_{i=1}^{n2} x_i^{dea} + \sum_{i=1}^{n3} x_i^{rev} = 1 \tag{14}$$

Let us denote these steady-state values by x_{*ss}^{*}. Note that system (11) with its equation for activation replaced by (14) has to be solved for each new parameter vector in the search procedure.

We have used the following initial conditions for system (11):

$$x_i^{act}(0) = u_{ini} x_{i\,ss0}^{act} \quad i=1 \ldots n1 \tag{15a}$$

$$x_i^{dea}(0) = u_{ini} x_{i\,ss0}^{dea} \quad i=1 \ldots n2 \tag{15b}$$

$$x_i^{rev}(0) = u_{ini} x_{i\,ss0}^{rev} \quad i=1 \ldots n3 \tag{15c}$$

$$x_i^{elo}(0) = u_{ini} x_{i\,ss0}^{elo} + (1 - u_{ini}) \frac{k_{spl} x_0^{spl}}{k_{elo} n5} \quad i=1 \ldots n4 \tag{15d}$$

$$x_i^{spl}(0) = u_{ini} x_{i\,ss0}^{spl} + (1 - u_{ini}) \frac{x_0^{spl}}{n5} \quad i=1 \ldots n5 \tag{15e}$$

$$x_i^{pro}(0) = u_{ini} x_{i\,ss0}^{pro} + +(1 - u_{ini}) \frac{k_{spl} x_0^{spl}}{k_{mat} n6} \quad i=1 \ldots n6 \tag{15f}$$

$$x_i^{mat}(0) = u_{ini} x_{i\,ss0}^{mat} + (1 - u_{ini}) \frac{k_{spl} x_0^{spl}}{k_{keg} n7} \quad i=1 \ldots n7 \tag{15g}$$

With u_{ini} defined as above, and the value x_0^{spl} is an unknown parameter to be fitted to the data.

The variety of multi-step models examined

In this paper we consider the following multi-step models

n = 1: $n_1 = n_2 = n_3 = n_4 = n_5 = n_6 = n_7 = 1$, $n_1^0 = 1$

n = 3: $n_1 = 3$, $n_2 = n_3 = 1$, $n_4 = n_5 = n_6 = n_7 = 5$, $n_1^0 = 1$

n = 5: $n_1 = n_2 = n_3 = n_4 = n_5 = n_6 = n_7 = 5$, $n_1^0 = 2$

n = 10: $n_1 = n_2 = n_3 = n_4 = n_5 = n_6 = n_7 = 10$, $n_1^0 = 4$

n = 20: $n_1 = n_2 = n_3 = n_4 = n_5 = n_6 = n_7 = 20$, $n_1^0 = 8$

Fit procedure

We used the two time courses for the ligand activation and the time course for the pre-mature mRNA degradation to obtain the unknown parameters in our models. The mature mRNA degradation data (Fig. 2D) were employed for validation.

Model parameters

The parameter vector p to be fitted with the three time courses (pre-mature and mature mRNA from the ligand activation and pre-mature mRNA from the degradation experiment) is for the simple model (n = 0) given by

$$p_s = (k_{ini}, k_{mat}, k_{deg}, x_0^{pro}) \tag{16}$$

for the two-step promoter activation model n=2 by

$$p_t = (ktf, kpol, kini, kmat, kdeg, x_0^{pro}) \tag{17}$$

and for the multi-step models (n = 1, 3, 5, 10, 20)

$$p_s = (k_{act}, k_{dea}, k_{rev}, k_{ini}, k_{elo}, k_{spl}, k_{mat}, k_{deg}, x_0^{spl}) \tag{18}$$

The initial guesses for the parameter search are based on the rough manual fit of the experimental data. They are given by

$$p_s = (\frac{1}{10}, \frac{1}{50}, \frac{1}{120}, 2.68) \tag{19}$$

$$p_t = (\frac{1}{7.5}, \frac{1}{2.5}, 1, \frac{1}{25}, \frac{1}{120}, 2.68) \tag{20}$$

$$p_m = (\frac{n1}{35}, \frac{n2}{10}, \frac{n3}{15}, 1, \frac{n4}{5}, \frac{n5}{10}, \frac{n6}{20}, \frac{n7}{120}, 2.68) \tag{21}$$

The initial guesses are then used to define the boundaries of the search domain used in the fitting procedure, which uses multiple initial parameter vectors. These search boundaries are $[\frac{k}{4}, 4k]$ for any kinetic constant k, and $[\frac{2x_0}{3}, \frac{4x_0}{3}]$ for any initial concentrations x_0.

Model observables

We denote the observables by (o^{pR} (t, p, u_{ini}), o^{mR} (t, p, u_{ini})) for the pre-mature mRNA and the mature mRNA respectively. For the simpler models (n = 0 and n = 2) the model observables are

$$o^{pR}(t, p, u_{ini}) = x^{pro}(t, p_{s/t}, u_{ini}) \tag{22a}$$

$$o^{mR}(t, p, u_{ini}) = x^{mat}(t, p_{s/t}, u_{ini}) \tag{22b}$$

and for the multi-step models (11a-g)

$$o^{pR}(t, p, u_{ini}) = \sum_{i=1}^{n5} x^{spl}(t, p_m, u_{ini}) \tag{23a}$$

$$o^{mR}(t, p, u_{ini}) = \sum_{i=1}^{n7} x^{mat}(t, p_m, u_{ini}) \tag{23b}$$

Object function

The distance measures per data set are

$$V^{l,pR}(p) = \sum_{i=1}^{N^l} \sum_{j=1}^{n_i^{l,pR}} (o^{pR}(t_i^l, p, 1) - ed^{l,pR}(t_i^l, j))^2 \quad (24a)$$

$$V^{l,mR}(p) = \sum_{i=1}^{N^s} \sum_{j=1}^{n_i^{l,mR}} \left(\frac{o^{mR}(t_i^l, p, 1) - ed^{l,mR}(t_i^l, j)}{10}\right)^2 \quad (24b)$$

$$V^{d,pR}(p) = \sum_{i=1}^{N^d} \sum_{j=1}^{n_i^{d,pR}} (o^{pR}(t_i^d, p, 0) - ed^{d,pR}(t_i^d, j))^2 \quad (24c)$$

$$V^{d,mR}(p) = \sum_{i=1}^{N^d} \sum_{j=1}^{n_i^{d,mR}} \left(\frac{o^{mR}(t_i^d, p, 0) - ed^{d,mR}(t_i^d, j)}{10}\right)^2 \quad (24d)$$

where N denotes the number of time-points, n the number of data-points per time point, and ed the experimental data; the upper-indices stand for l-ligand addition experiment, d-degradation experiment, pR-pre-mature mRNA, mR-mature mRNA. The object function we use for the fit procedure is

$$V(p) = V^{l,pR}(p) + V^{l,mR}(p) + V^{d,pR}(p) \quad (25)$$

The first part (24a) compares the model with the pre-mature mRNA data of the ligand activation experiment, the second part (24b) with the mature mRNA data of the ligand activation experiment, the third part (24c) with the pre-mature mRNA of the degradation experiment, while (24d) is be used to evaluate the quality of the validation with mature mRNA decay time course and is not involved in the objective for the fitting therefore. We have scaled the two 'mature' terms with 10 to take the difference of the concentration values into account. Please note that the parameter vector p, for which this object function V is minimal, is a Weighted Least Squares Estimate. For it to be a MLE (1) the assumption is needed that the data-errors for all time points are independent and normally distributed with (scaled) variance 1.

Search

We employed a standard global search algorithm, called Controlled Random Search (CRS) (2). The main idea behind this algorithm is that, starting with an initial collection of parameter vectors, CRS repeatedly draws a new parameter vector that replaces a vector in the collection if its data fit is better. The CRS method starts with taking a random set of parameter vectors inside a search domain by drawing random values from the uniform distribution (confined by *D)* for each parameter value for each vector. Then the corresponding values of the object function are computed for each vector in the set. The bounds of represent the *a priori* limits for the parameters.

From this list of n_Q vectors, a new vector is created using the rule

$$p_{new} = 2\bar{p}_{rand} - p_{rand} \quad (26)$$

where is a random vector from the domain, and is the average of a random subset of vectors in the domain. To ensure that the new vectors are selected with equal preference over the logarithmic space, equation (26) is modified element wise to

$$p_{new} = 10^{\log(2\bar{p}_{rand}) - p_{rand}} \tag{27}$$

If $V(p_{new}) < \max(V(p))$, and $p_{new} \in D$, the parameter vector with the highest object function is replaced by

$$\bar{p} | \{S(\bar{p}) = \max(S(p))\} \rightarrow p_{new} \tag{27}$$

By repeating this, the worst fitting parameter vectors are removed continuously and replaced by ones with a better fit. Eventually, the points will form a cloud that gets denser and denser. The algorithm stops when $\max(V(p)) \leq s_c \min(V(p))$ with s_c the stop criterion. So the worst fit in the remaining collection has an at most % larger V value than that of the best fit. We used the following values: $s_c = 1.01$, $n_Q = 300$. The frequence with which a parameter value was found in new parameter vectors during the fitting is shown in Fig. S7.

The resulting optimal parameters are shown in Table S6.

Statistical and sensitivity analysis

Residual analysis

The first step in our *a posteriori* analysis is based on the residual values only. With this we can test whether we should reject the hypothesis that our mathematical model with the obtained parameter vector $\hat{p}$ is an acceptable description of the data.

There are two types of residual test:

Size-based. Here we test the probability that the obtained $V(\hat{p})$ lies in the χ^2 distribution corresponding to our number of data-points and number of unknown parameters: χ^2 -test $T_{\chi^2} = V(\hat{p}) \in \chi^2(\text{df})$. If the test value $T > \delta(\alpha)$, the hypothesis is rejected. The δ is the value such that cumulative distribution function CDF ($\chi^2(\text{df})) = 1-\alpha$, with α the confidence level and df the degrees of freedom. The df is defined as df = N-m, where N is the number of data points fitted and m the number of parameters under assumption that the parameters are independent.

Correlation-based. Here the probability is tested that the residuals are uncorrelated: runs-test $T_{run} = \frac{R_u - N/2}{\sqrt{N/2}} \in N(0,1)$, where R_u is a function of sign changes in $e(\hat{p})$, which is the vector of differences per timepoint between the model results and the average of the data points. Again if the test value $T > \delta(\alpha)$, the hypothesis is rejected.

For all models 95% χ^2 -tests are passed, all pass the 95% runs-test with exception of n=0 model (both described above; the interval for 5% confidence interval is (-1.96,1.96)).

Model discrimination

Because models n = 0 and n = 2 have different number of parameters compared to multistep models with n = 1,3,5,10 and 20 we calculated Akaike Information Criterion (AIC) in order to objectively access which models describe the data better. The AIC is defined as:

$$AIC = \frac{V(\hat{p})}{N} + \frac{2\, d_{mc}}{N} \quad (28)$$

where d_{mc} is the model complexity, defined as the number of independent parameters. For the n = 0 model $d_{mc} = 4$, while for the n = 2 model $d_{mc} = 5$ and for multi-step models $d_{mc} = 8$ (rate constants describing mRNA production rate are co-dependent for all n>0 models, so the number of independent parameters is the total number of parameters minus 1). According to the AIC criterion all the multi-step models are statistically more likely than the simpler n = 0 and n = 2 models (Table S7).

Since all multistep models have similar properties according to the statistical tests we can try to further discriminate according to the value of the object function – smaller value means better overall fit. Additionally, smaller run-test values are also preferable. Keeping this in mind the better models seem to be n = 1, n = 3 and n = 5, although the differences between all multi-step models are not dramatic.

Sensitivity analysis (n = 5 model)

For local analysis the pairwise covariance coefficient of the parameters (Table S8) was calculated. First the sensitivity matrix *J* was determined by perturbing each parameter by 0.01% in both directions and calculating the corresponding sensitivity coefficient of $V(\hat{p})$at each data point, resulting in a 396 x 8 matrix. The covariance matrix is computed as $cov = (J^T J)^{-1}$and the correlation coefficient between i-th and j-th parameter as $cov_{ij} = \frac{cov_{ij}}{\sqrt{cov_{ii} cov_{jj}}}$. Further, parameter dependent and independent 95% confidence intervals were calculated (Table 2). For global independent parameter sensitivity analysis, parameters were sampled one parameter at a time from a uniform distribution with limits four times lower and higher than the best-fit value. This resulted in N = 3,000 new parameter vectors with a single parameter perturbed. Then for each new parameter vector fold change of the object function compared the best fit was calculated. The results are plotted in Fig. S9.

Additionally, the co-dependent parameter analysis was done by sampling the three out of four co-dependent promoter activity rate constants (k_{act}, k_{ini} and k_{rev}) from respective uniform distributions. The ranges were as follows: 0.033^{-6} min^{-1} for k_{ini}, 0.025^{-5} min^{-1} for k_{act} and k_{rev}. The fourth constant (k_{dea}) was adjusted to keep the mRNA production rate at a fixed value determined by the following relationship:

$$\frac{k_{act}\, k_{rev}\, k_{ini}}{k_{act}\ k_{dea} + k_{act}\ k_{rev} + k_{rev}\ k_{dea}} = v_{mRNA\, prod} \quad (29)$$

The rate of mRNA production found in the fitted model was 0.238. With exception of cases when k_{act} deviates from the found by fitting by high percentage values the resulting fits were very close to the original fit (90% of fits have deviation less than 10% of the original).

Validation of the n = 5 model

To validate the n = 5 model the model output was compared to the mature mRNA decay data (Table S5). Since the mRNA decay was measured after 180 min of ligand treatment and addition of the inhibitor results in inhibition of initiation at the TSS the following initial conditions were used:

$$x_i^{act}(0) = 0 \quad i=1...n1 \tag{30a}$$

$$x_i^{dea}(0) = 0 \quad i=1...n2 \tag{30b}$$

$$x_i^{rev}(0) = 0 \quad i=1...n3 \tag{30c}$$

$$x_i^{elo}(0) = x_i^{elo}(180) \; i=1...n\,4 \tag{30d}$$

$$x_i^{spl}(0) = x_i^{spl}(180) \; i=1...n5 \tag{30e}$$

$$x_i^{pro}(0) = x_i^{pro}(180) \; i=1...n6 \tag{30f}$$

$$x_i^{mat}(0) = x_i^{mat}(180) \; i=1...n7 \tag{30g}$$

where the $x_i^n(180)$ is the value of the x_i^n after 180 min of ligand addition. The promoter variables are set to zero as initiation has been inhibited. Since the inhibitor used does not inhibit the polymerases that have escaped the promoter all the mRNA species are still present after the addition of the inhibitor. The value of the resulting objective function $V^{d,mR}(\hat{p})/N^{d,mR}$ was 0.48 which is comparable to that of the model fit in the ligand activation experiment.

Global analysis of the independent parameter sensitivity of the validation fit was carried out in the same manner as the model fit. The results indicate that the validation has the same sensitivity as the model fit with the exception of being insensitive to k_{spl} value (Fig. S10).

Refitting the ligand induction time-course after splicing inhibition

The additional data after splicing inhibition was treated in the same way as the other datasets (Table S9).

For re-fitting the model the induction factor f was adjusted to the experimentally observed 1.42 value calculated as the mean of the last five pre-mRNA data points (150-180 min; the mature mRNA does not reach a steady state level in the duration of the experiment). The same observables were used as in the original fitting procedure. The distance measures per dataset were:

$$V^{s,pR}(p) = \sum_{i=1}^{N^s} \sum_{j=1}^{n_i^{s,pR}} \left(o^{pR}\left(t_i^s, p, 1\right) - ed^{s,pR}\left(t_i^s, j\right)\right)^2 \tag{31a}$$

$$V^{s,mR}(p) = \sum_{i=1}^{N^s} \sum_{j=1}^{n_i^{s,mR}} \left(\frac{o^{mR}(t_i^s,p,1) - ed^{s,mR}(t_i^s,j)}{5}\right)^2 \quad (31b)$$

The object function was defined as sum of the distance measures:

$$V^s(p) = V^{s,pR}(p) + V^{s,mR}(p) \quad (32)$$

Each single parameter was allowed to change within 4-fold of its original value and the model was fitted anew to the perturbed dataset (Table S9).

In the second round (run 2) in Table S19 the changed k_{spl} (which produced the best fit in run1) was inserted into the model and the model was fitted varying each of the remaining rate constants by 4-fold. The list of the factors by which constants were adjusted and of the residuals is presented in Table S10.

The sensitivity of the refit to the individual constants perturbations performed in the same way as in the a posteriori analysis of the original model is presented in Fig. S14.

Finally, the dependence of the prediction quality on the specific parameter values was tested. All but the adjusted parameters were sampled independently according to the same procedure as for the parameter sensitivity analysis of the original fit. Then for each new parameter vector the fold changes of the object function compared to the optimal parameter vector were calculated for both (the original and perturbed) models. The relationship between fold change in the object function of the original model and the perturbed is plotted Fig. S13 (blue). Same type of sensitivity analysis was done by sampling co-dependent promoter activity rate parameters (k_{act}, k_{dea}, k_{ini} and k_{rev}) according to same procedure as for the original fit (Fig. S13, violet).

Figures

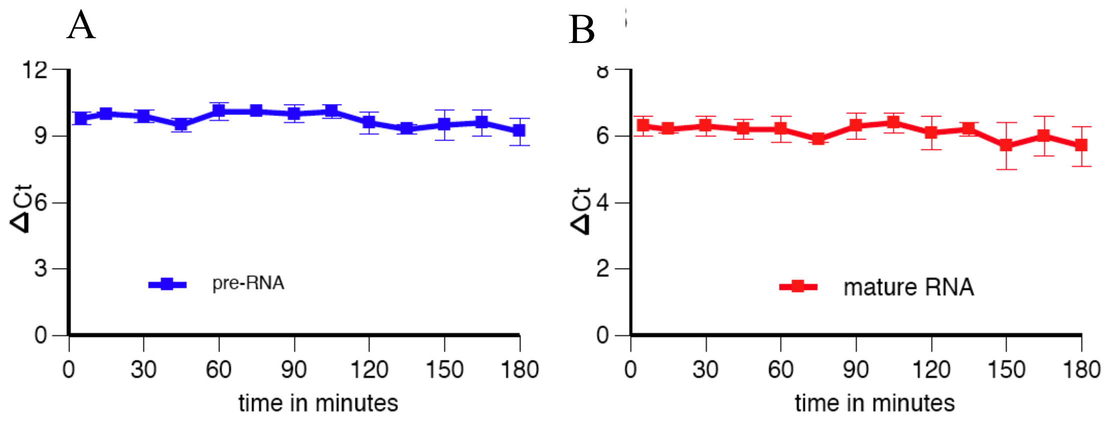

Fig. S1: Invariance of Ct values. The values of ΔCt (Ct_{ADRP}-Ct_{RPLP0}) for DMSO-treated samples pre-mRNA (A) and mature RNA (B) are displayed over time. Data points represent averages from three experiments. Bars represent standard deviation.

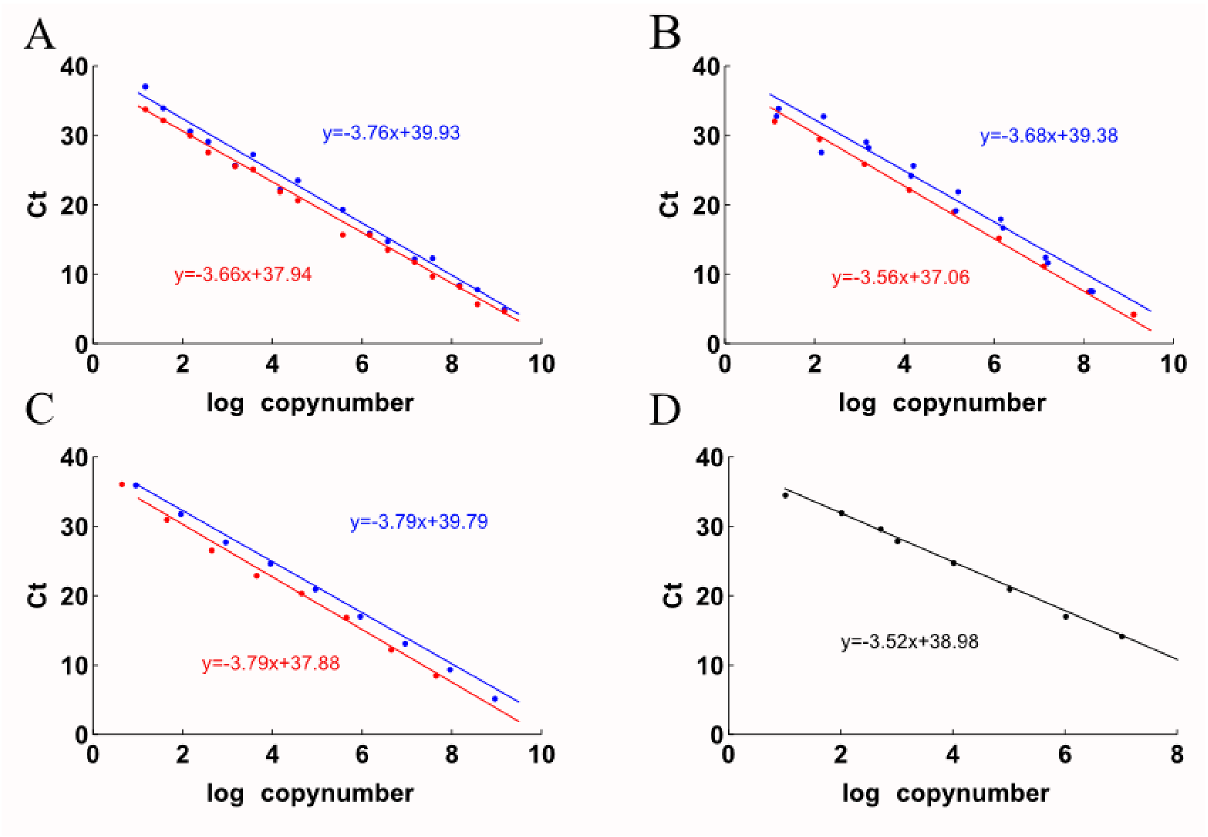

Fig. S2: Standard curves for the absolute mRNA quantification. qPCR was performed on known amounts of cDNA. The resulting Ct values were plotted against absolute copy number calculated from the measured DNA concentration and individual molecular weights of the

standard fragments. For the *ADRP* gene two independent experiments were performed and fitted separately resulting in the maximal (blue) and minimal (red) fits used to estimate the method error. The fit to all data points (not shown) was used to calculate the average copy number. The standard curves were obtained for intron 1-exon1 (A), exon1-exon3 (B), exon5-exon6 (C) and the *Barnase* control gene (D) fragments. Log refers to the 10-based logarithm where Ct is, as usual 2-log based. The theoretical value of the direction coefficient is thereby $-{}^{2}\log 10 = -3.32$.

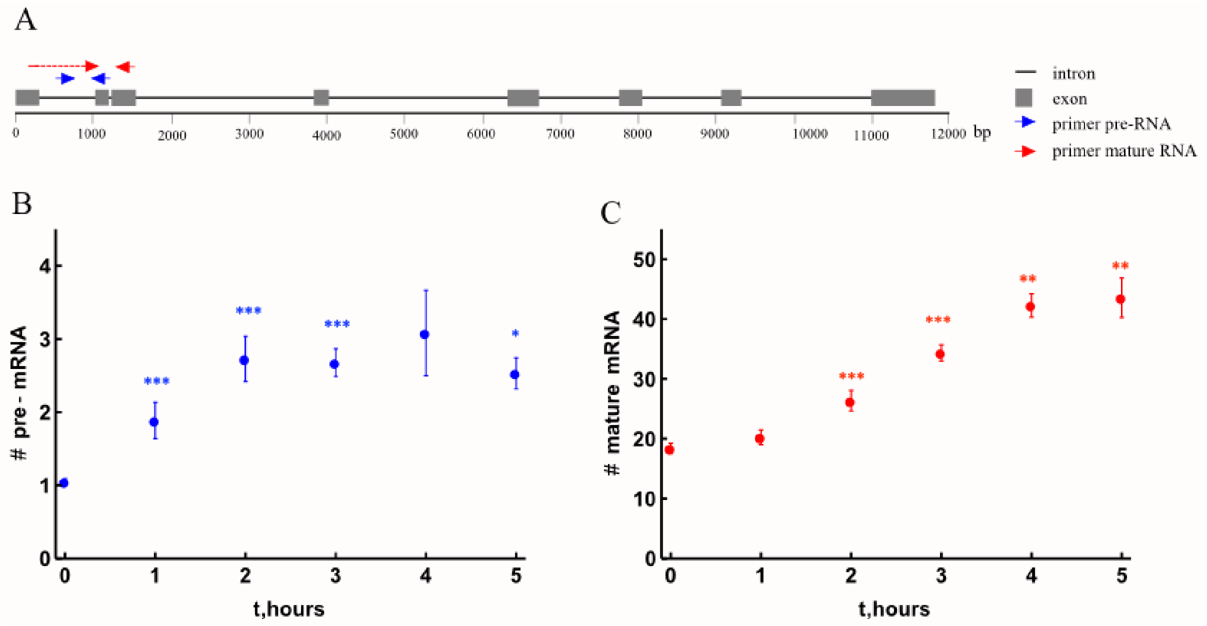

Fig. S3: Up-regulation of the *ADRP* gene upon PPARδ ligand treatment. (A) Location of the used qPCR primers in relation to the introns (lines) and exons (boxes) of the human *ADRP* gene. The PCR primer pair marked in blue monitors the presence of the first intron, as the right primer lies on the exon-intron boundary. After the splicing step in which this intron is excised, this primer pair will no longer give a PCR product. Therefore this primer pair operationally defines the 'pre-mRNA' (B), corresponding to the sum of all states of mRNA that still have to be spliced on the first intron. The PCR primer pair indicated in red targets the border of exon 1 and 2 and therefore gives a product only when exon 1 has been excised by splicing. In combination with only measuring polyadenylated fraction of mRNA this primer pair operationally defines what we shall call 'mature' mRNA (C). HepG2 cells were treated for indicated times with 100 nM GW501516, the data was normalized to housekeeping gene expression and fold inductions were calculated in comparison to vehicle (DMSO) control. Data points indicate the means of at least three independent experiments corrected for outliers using the MAD method (3). Error bars represent the standard error of the mean. Two-tailed, paired Student's t-tests were performed to determine the significance of the ligand-dependent regulation of *ADRP* RNA in reference to vehicle (* $p<0.05$, ** $p<0.01$, *** $p<0.001$)

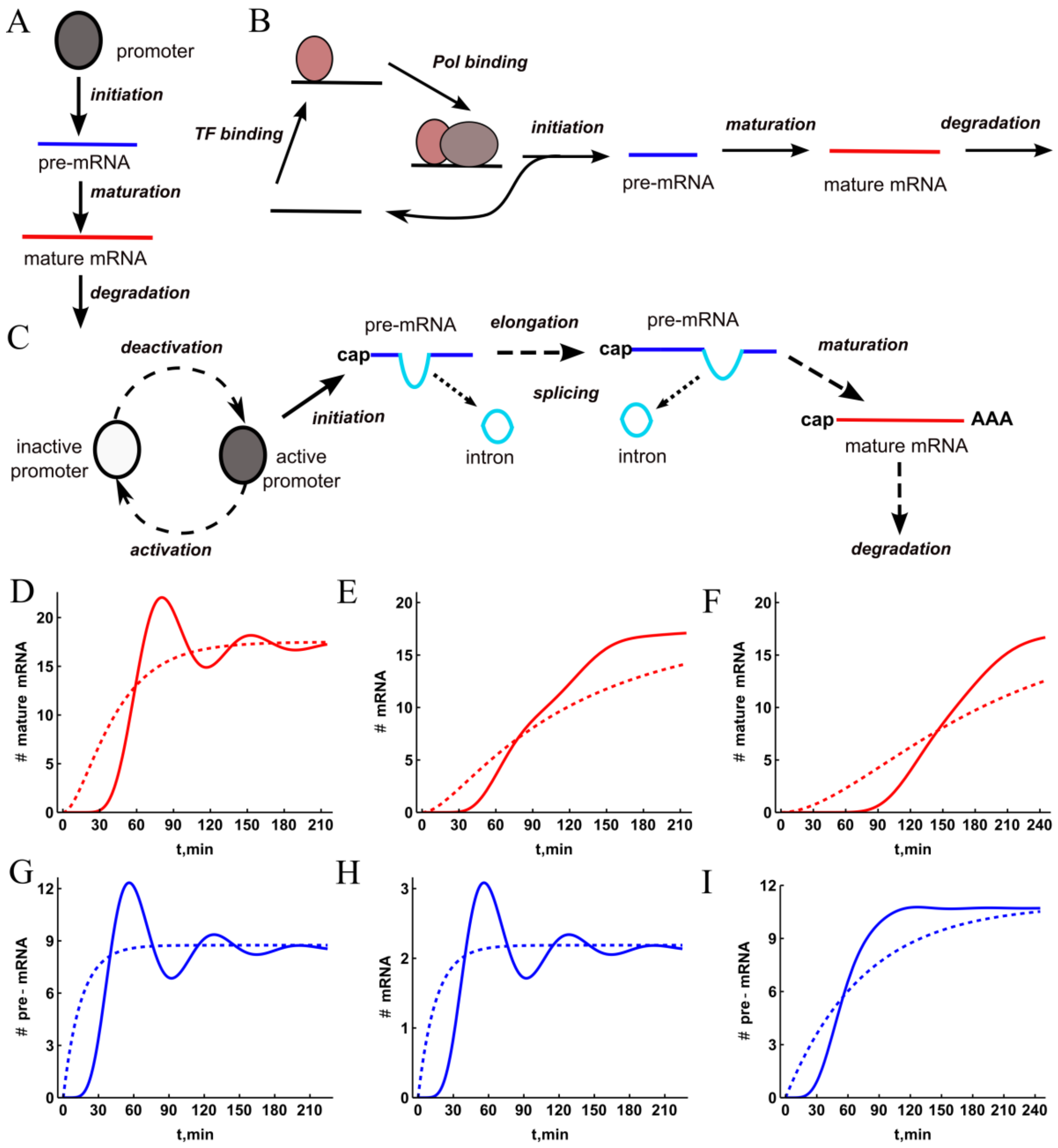

Fig. S4: Alternative models of mRNA dynamics. (A) A highly simplified model of a constitutively active promoter and first-order mRNA elongation and degradation process is shown. (B) A more sophisticated model that includes two step promoter activation (C) A realistic model that takes into account the mRNA dynamics cycle, as well as the multi-step nature of mRNA maturation (including elongation and splicing) and degradation. The transcriptional dynamics cycle, maturation and degradation were each taken to have an arbitrary number of 15 irreversible reactions with equal rates. The plots below show mature mRNA (D-F) and pre-mRNA (G-I) induction time courses for the two different models (solid line; refined model C, dotted line: simplified model A) under various parameter regimes: (D, G) when promoter cycling time (T_{cyc}) exceeds the degradation time (T_{deg}) and maturation time (T_{mat}), (E, H) when $T_{cyc} < T_{deg}$ and $T_{cyc} > T_{mat}$, and (F, I) when $T_{cyc} < T_{deg}$ and $T_{cyc} < T_{mat}$. All models start with initial conditions corresponding to zero transcription activity (gene regulatory region in inactive state) and same steady state for mature mRNA.

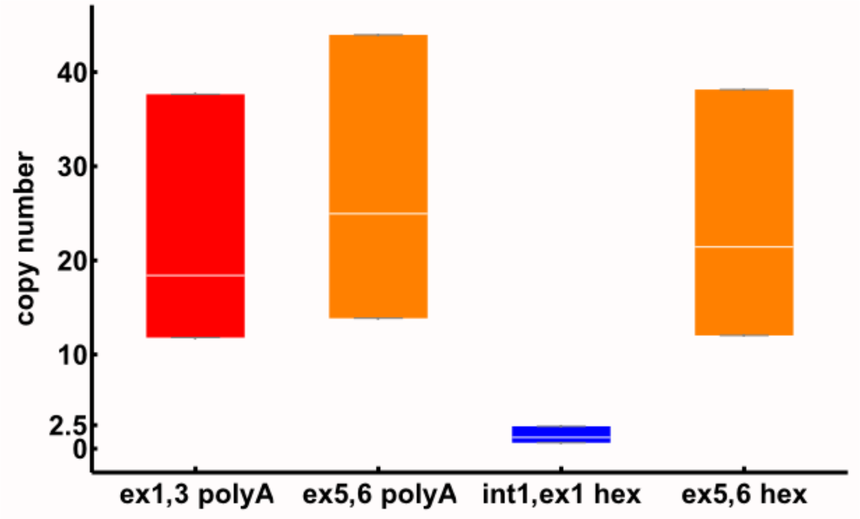

Fig. S5: Copy numbers of different mRNA species. Copy numbers of pre-mRNA (blue) and mature mRNA (red and orange) were calculated based on qPCR data (Figs. S3B and C). Averages are shown as horizontal lines (based on the average data fit of all the points in Fig. S2) and the maximum-minimum estimation range (based on the blue and red data fits in Fig. S2, respectively) is indicated by the bars.

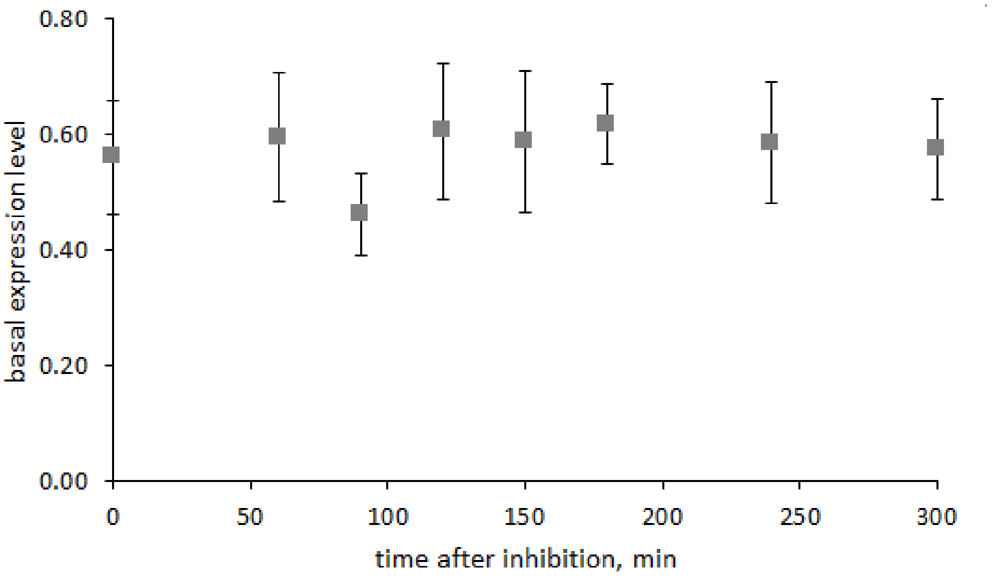

Fig. S6: *GAPDH* degradation rates. HepG2 cells were treated with 100 nM GW501516 for 3 h and then new mRNA synthesis was blocked by the application of 50 µM DRB. The relative levels of mature RNA of the very stable gene *GAPDH* were measured in relation to the housekeeping gene *RPLP0* by qPCR. Data points indicate the means of at least three independent experiments; error bars represent the standard error of the mean.

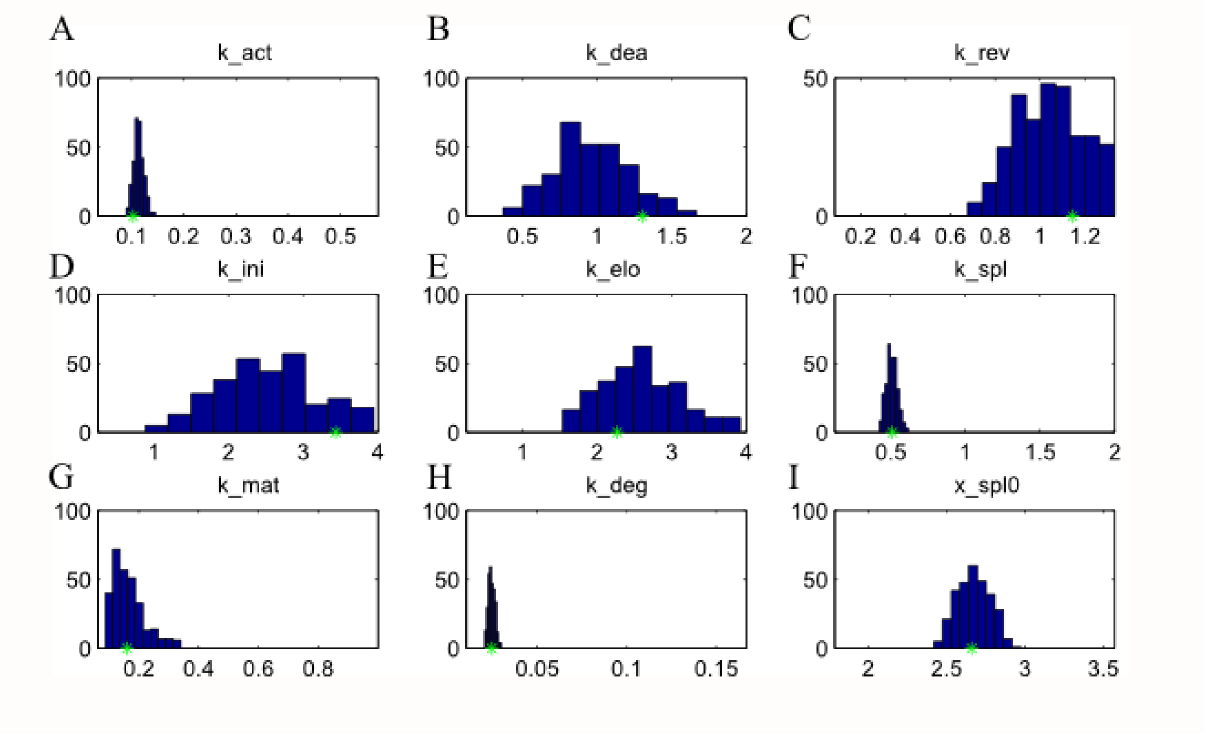

Fig. S7: The parameter values explored in the fitting procedure of the n = 5 model. During the fitting procedure new parameter vectors are created according to the following rule $p_{new} = 10^{\log(2\bar{p}_{rand}) - \log(p_{rand})}$. where $\bar{p}_{rand}$ is the average of all initial vectors created by drawing from uniform random distributions. The parameter values are described by distributions plotted for all rate constants (A-H) and for pre-mRNA decay initial conditions (J). The green star indicates the optimal value of the parameter.

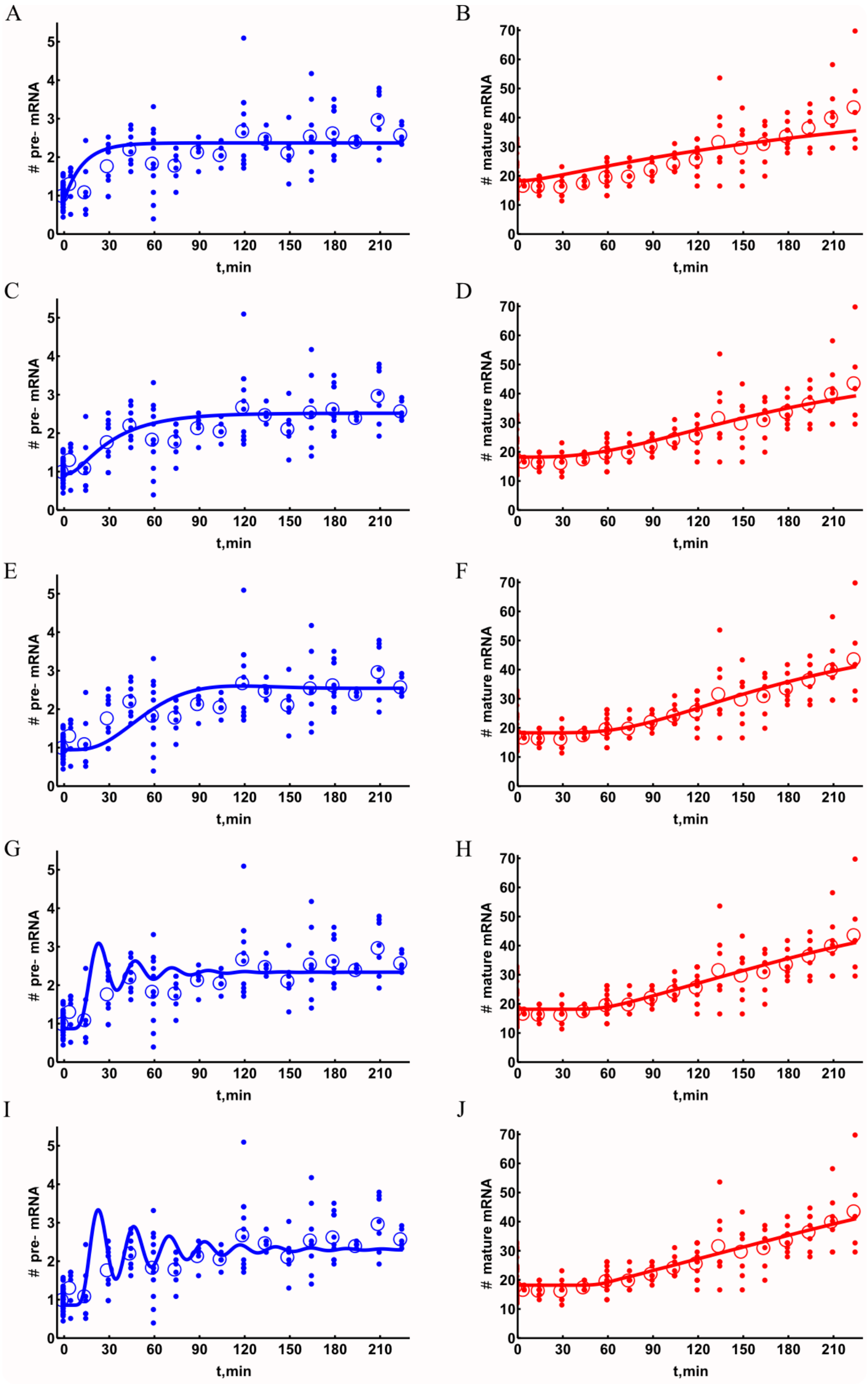

Fig. S8: The fitted mRNA induction time course data for four different models: n = 0 (A, B), n = 1 (C, D), n = 3 (E, F), n = 10 (G, H) and n = 20 (I, J). The time course for pre-mRNA is shown on the left and for the mature mRNA on the right. The fit for the n = 5 model is shown in Fig. 2. Small filled circles present all data points used for fitting, empty circles represent the calculated mean.

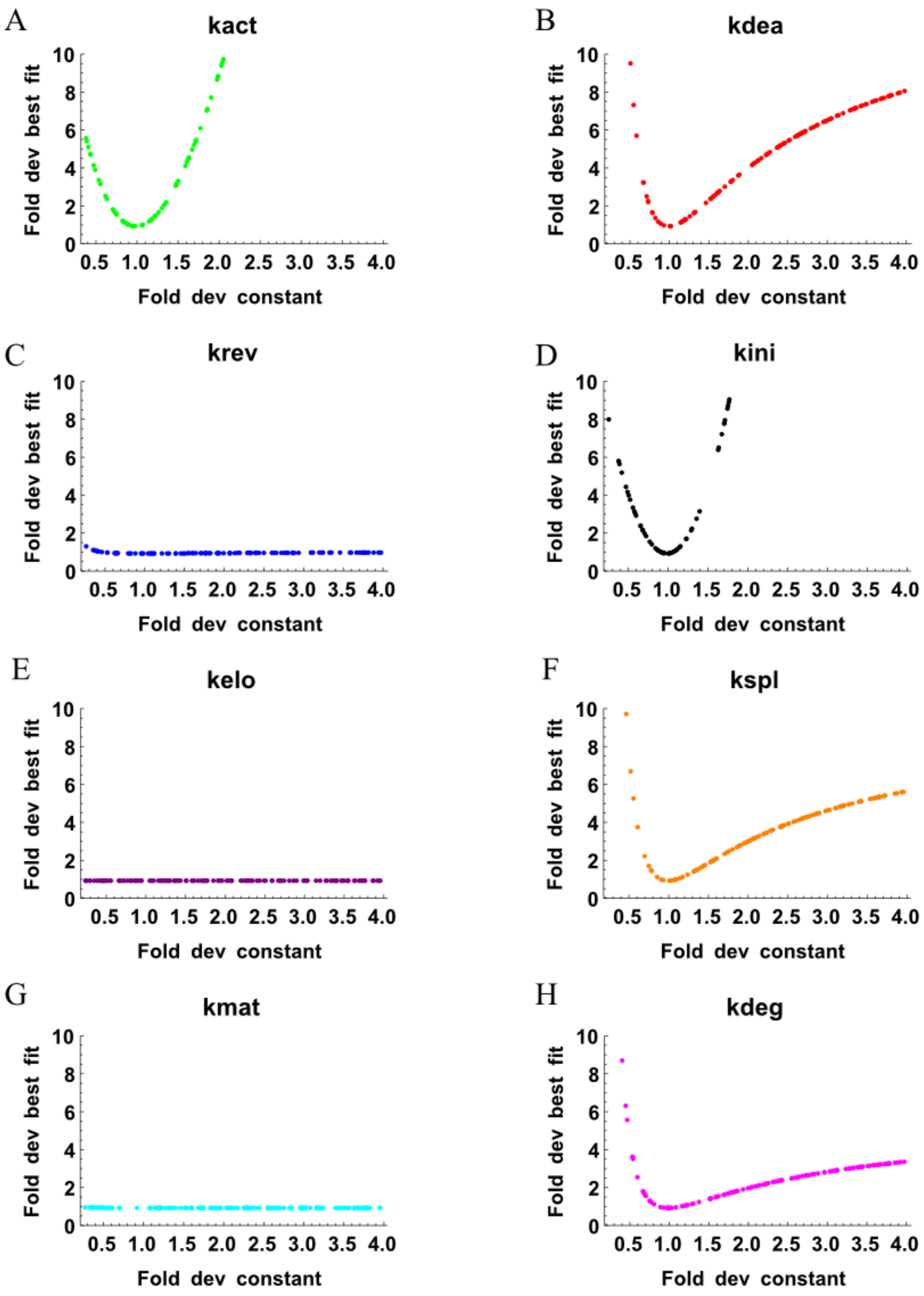

Fig. S9: ***A posteriori*_analysis of the model n = 5 fit sensitivity to the independent parameter perturbation.** The fold deviation of the object function from the best fit $V(\hat{p}_i)/V(\hat{p}_i^{pr})$ (where $\hat{p}_i$ is the perturbed parameter) is plotted against the fold deviation of the parameter perturbation $\frac{\hat{p}_i}{\hat{p}_i^{pr}}$.

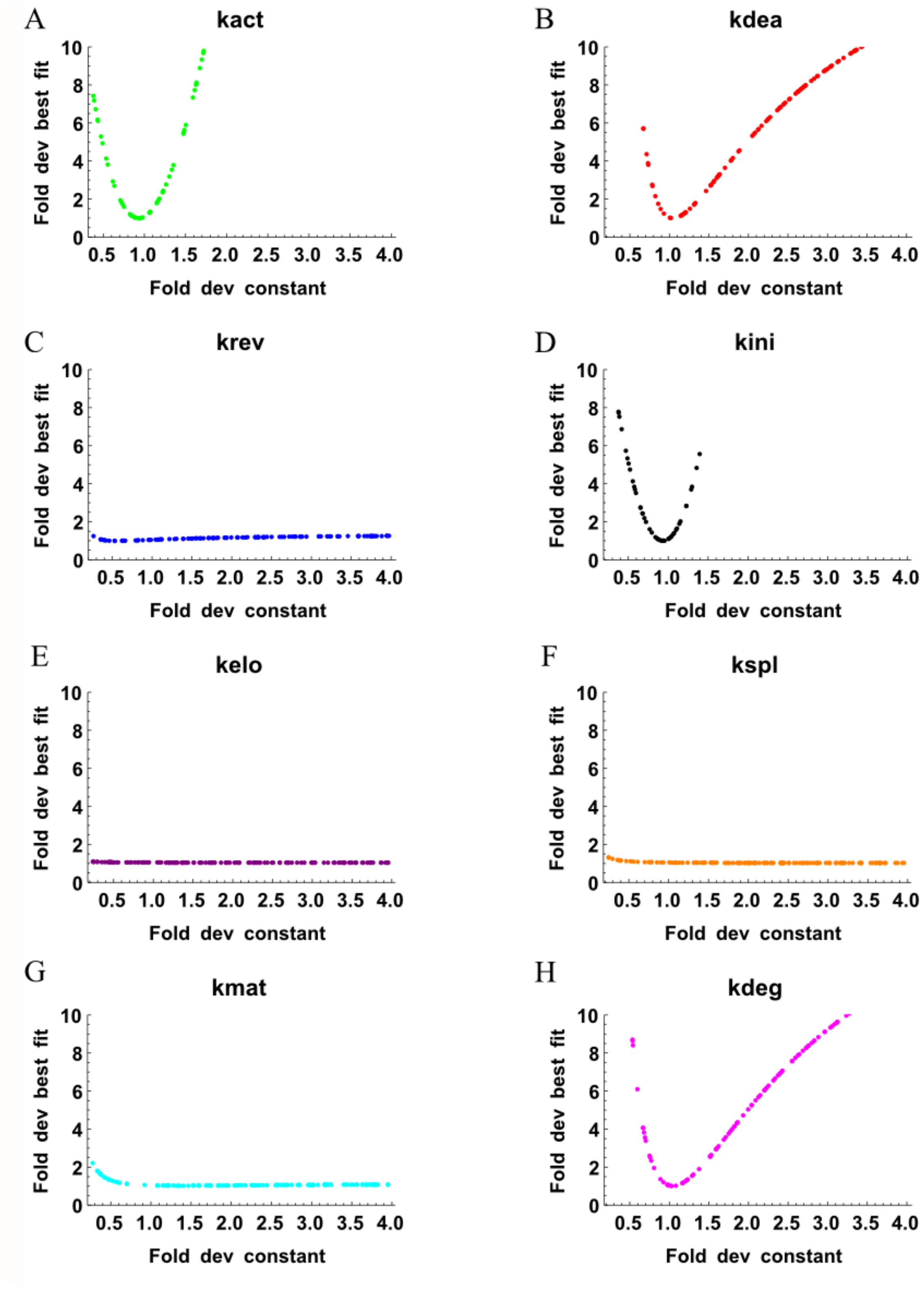

Fig. S10: Analysis of the model refit (after splicing inhibition) sensitivity to the independent parameter perturbation The fold deviation of the object function from the best fit $V(\hat{p}_i)/V(\hat{p}_i^{pr})$ (where $\hat{p}_i$ is the perturbed parameter) is plotted against the fold deviation of the parameter perturbation $\frac{\hat{p}_i}{\hat{p}_i^{pr}}$.

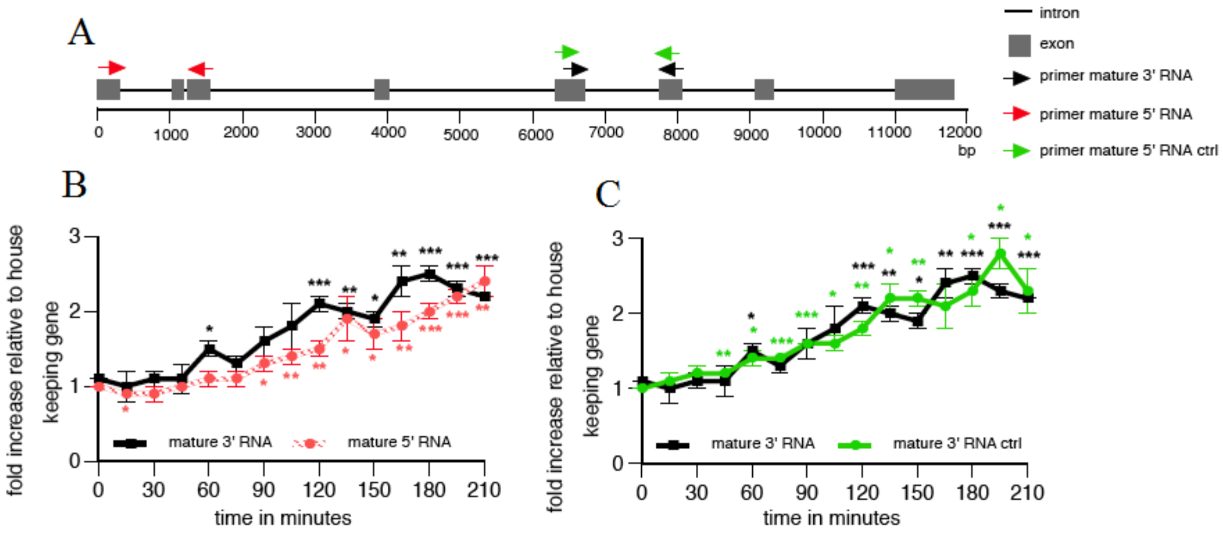

Fig. S11: Estimation of a degradation mechanism and primer set validation. Human HepG2 cells were stimulated with 100 nM GW501516, total RNA was collected and qPCR was performed using the polyadenylated RNA fraction as template. (A) Primer localization relative to the introns (lines) and exons (boxes) of the human *ADRP* gene. (B) In order to determine 5' degradation, primers located in exons 1,3 (red) were used for qPCR. The results were compared with that obtained by qPCR with primers located in exons 5,6 (black). (C) Control experiment with both primer sets in exons 5 and 6. Data are presented as fold induction of ligand over vehicle (DMSO) treatment. Each data point represent the mean of at least three biological replicate samples corrected for the outliers with MAD method, error bars represent the standard error of the mean. Two-tailed, paired Student's t-tests were performed to determine the significance of the ligand-dependent regulation of *ADRP* RNA in reference to vehicle (* $p<0.05$, ** $p<0.01$, *** $p<0.001$).

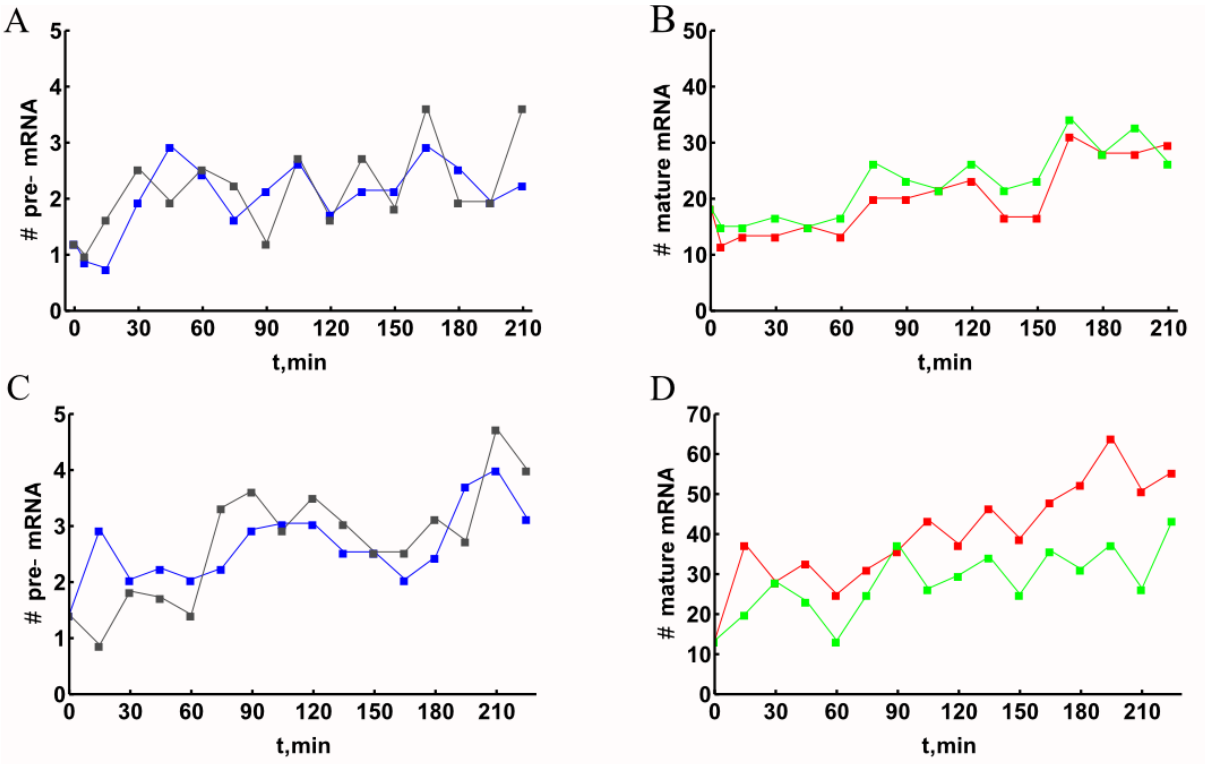

Fig. S12: Splicing inhibition. HepG2 cells were incubated simultaneously with 100 µM isoginkgetin and 100 nM GW501516 (A,B) or pre-treated for 3 h with 100 µM isoginkgetin before adding GW501516 for the indicated time (C,D). The control was mock treatment with vehicle (DMSO) instead of inhibitor/ligand. The levels of pre-mRNA (A,C) and mature mRNA (B,D) were measured by qPCR and the copy numbers were calculated. The data points represent the mean of two biological repeats.

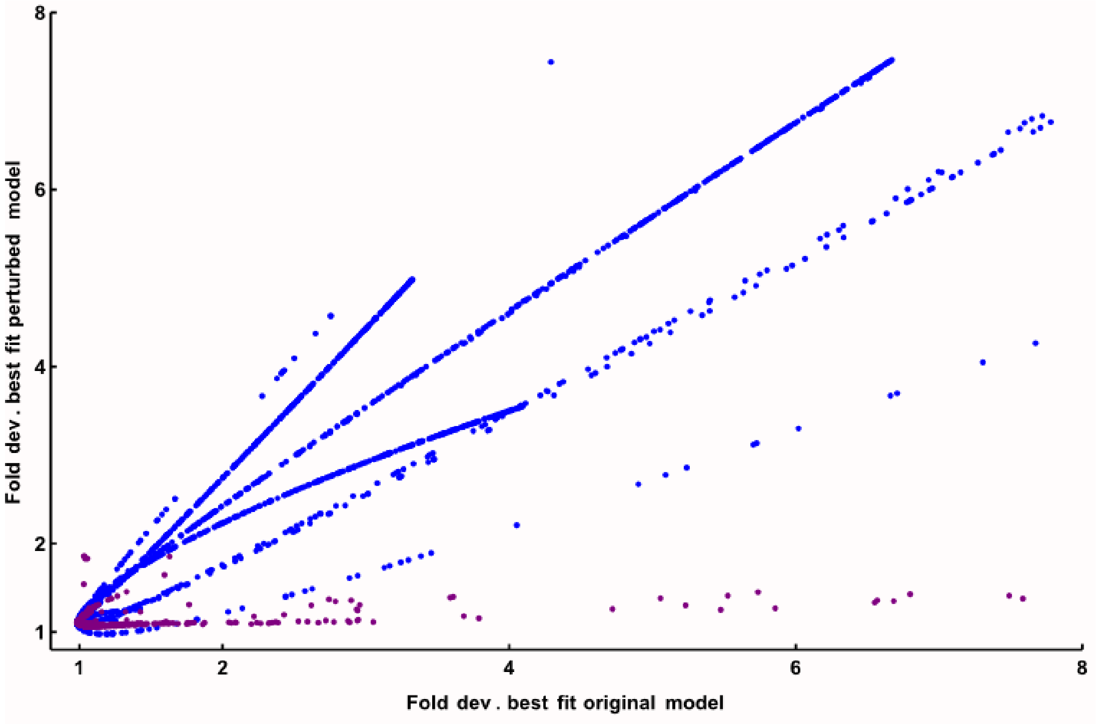

Fig. S13: The effects of parameter perturbation on the splicing inhibition data fitting. Independent parameter changes were sampled for all parameters (except k_{spl} and k_{deg}) at random using the same procedure as for the parameter sensitivity analysis described above. Co-dependent changes in the promoter parameters (k_{act}, k_{ini}, k_{dea} and k_{rev}) were sampled as well according to the described procedure. The respective fold deviation from the best fits of the original and perturbed models were calculated and plotted against each other. The results obtained with independent parameter sampling are shown in blue; the ones obtained with dependent parameter sampling are in purple.

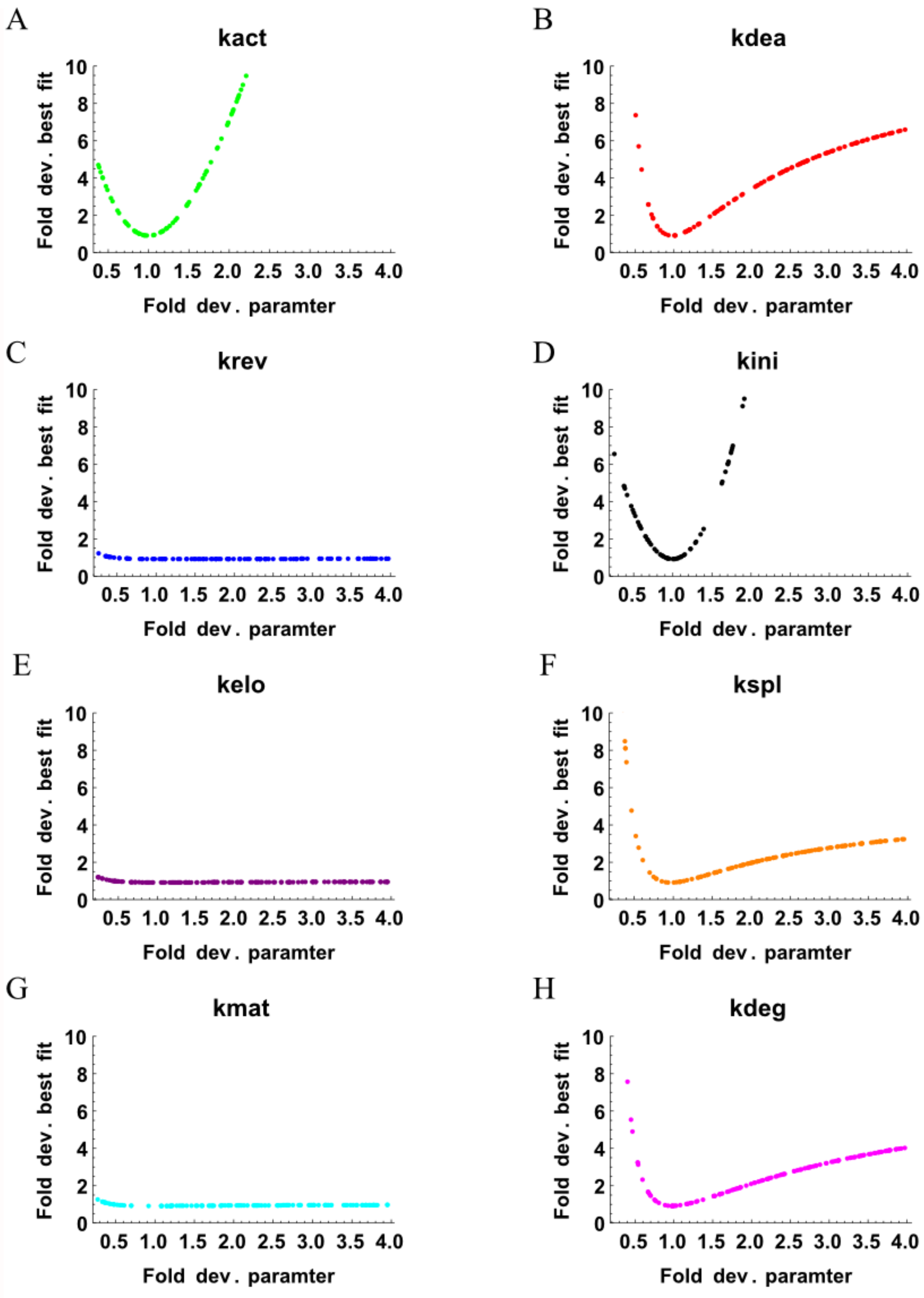

Fig. S14: Analysis of the model validation fit sensitivity to the independent parameter perturbation. The fold deviation of the object function from the best fit $V(\hat{p}_i)/V(\hat{p}_i^{pr})$ (where $\hat{p}_i$ is the perturbed parameter) is plotted against the fold deviation of the parameter perturbation $\frac{\hat{p}_i}{\hat{p}_i^{pr}}$.

Tables

Gene fragment	Sequence
**mature RNA 5’ F**	GCA-GTC-CGT-CGA-TTT-CTT-TC
**mature RNA 5’ R**	ACC-GTT-CTC-TGC-CAT-CTC-AC
**mature RNA 3’ F**	GTA-GAA-CAG-TAC-CTC-CCT-CTC
**mature RNA 3’ R**	CGT-GAC-TCA-ATG-TGC-TCA-G
**mature RNA 3’ctrl F**	CAG-TGA-CTG-GCA-GTG-TGG-AG
**mature RNA 3’ctrl R**	ACG-GGA-GTG-AAG-CTT-GGT-AG
**pre-mRNA F**	TGG-AGA-GCT-GGA-GAG-AGG-AA
**pre-mRNA R**	GTT-GTG-GAT-CAA-CTG-CAA-CG
***RPLP0* F**	AGA-TGC-AGC-AGA-TCC-GCA-T
***RPLP0* R**	GTG-GTG-ATA-CCT-AAA-GCC-TG
***GAPDH* F**	CAT-GAG-AAG-TAT-GAC-AAC-AGC-CT
GAPDH **R**	AGT-CCT-TCC-ACG-ATA-CCA-AAG-T
**Barnase F**	AGC-CAA-CCA-CTG-AGG-ATC-TG
**Barnase R**	GTC-TGC-AAG-GTT-CCC-TTT-TG

Table S1: PCR primer pairs for qPCR of the human *ADRP* gene and house-keeping genes *RPLP0* and *GAPDH*. The annealing temperature was in all cases 60 °C. The primers for *ADRP* mature RNA 5’, mature RNA 3’, mature RNA 3’ctrl and pre-mRNA are color-coded in Figs. S6 and 11 red, black, green and blue, respectively.

	Model	k_{act}	k_{dea}	k_{ini}	k_{mat}	k_{deg}
$T_{cyc}>T_{mat}$	a*	0.167	0.500	4.000	1.000	0.500
$T_{cyc}>T_{deg}$	b*	-	-	0.583	0.067	0.033
$T_{cyc} > T_{mat}$	a	0.167	0.500	1.000	1.000	0.125
$T_{cyc} < T_{deg}$	b	-	-	0.146	0.013	0.008
$T_{cyc} < T_{mat}$	a	0.167	0.500	1.000	0.200	0.125
$T_{cyc} < T_{deg}$	b	-	-	0.146	0.013	0.008

Table S2: Parameter values used for alternative mRNA metabolism models simulation. These values were used for calculating the graphs presented in Fig. S4. a: simplified single step model, b: multi-step model for promoter cycle, maturation and degradation.

t	pre-mRNA											n
0	1.03	1.07	1.19	1.57	0.71	0.63	0.93	0.8	1.32	0.79	0.89	39
	0.84	1.03	1.2	1.52	0.46	1.55	0.92	0.94	1.11	1.19	1.07	
	0.91	1.02	0.89	1.21	0.63	0.72	0.59	0.95	1.59	1.02	1.17	
	1.21	1.29	1.3	1.23	1.37	1.35						
5	1.73	1.73	1.53	0.53	0.99	1.64						6
15	1.64	2.45	0.65	1.1	0.53	0.65	0.99					7
30	0.99	2.54	1.42	0.99	2.35	1.53	2.15	2.15	2.25			9
45	2.35	2.74	1.95	2.85	2.54	2.15	1.84	1.64				8
60	2.45	2.25	1.1	0.76	3.33	1.73	1.84	0.41	2.64	2.74	1.84	12
	1.53											
75	2.05	2.25	1.1	1.53	1.95	1.73	2.25					7
90	2.15	2.54	1.64	2.25	2.35							5
105	2.05	1.73	2.05	2.25	2.45							5
120	3.43	2.64	1.73	1.84	1.95	1.84	2.85	3.14	3.43	5.11	2.05	11
135	2.25	2.54	2.45	2.85	2.54							5
150	2.05	2.35	2.25	1.32	1.95	3.05						6
165	1.64	2.45	2.15	3.52	2.85	1.42	2.54	4.19				8
180	1.95	3.22	3.22	2.05	3.52	2.05	2.64	3.33	2.45	2.35		10
195	2.45	2.35	2.45	2.54								4
210	3.72	2.74	1.95	2.25	3.81	3.63	3.05					7
225	2.85	2.54	2.35	2.45	2.94							5
N=17												154

Table S3: pre-mRNA species copy numbers of the PPARδ ligand induction experiments. Calculated copy numbers of the experiments reported in Fig. 2A (pre-mRNA, observable 1). Time t is in minutes, n refers to the number of replica data points at the same time point and N is the number of time points examined.

t	mature mRNA											n
0	15.35	13.55	17.18	12.24	15.13	17.68	15.84	16.48	21.5	13.75	15.29	48
	16.48	13.55	14.36	19.22	12.01	14.13	17	15.74	15.42	18.55	12.92	
	14.42	18.9	30.37	12.52	15.77	20.08	31.6	13.52	17.07	18.98	27.95	
	12.86	18.32	32.46	12.21	12.16	12.21	16.04	17.39	23.18	30.95	24.67	
	23.86	24.11	25.13	33.14								
5	16.76	16.76	18.4									3
15	15.07	20.09	16.76	13.39	16.76	15.07	20.09	18.4	16.76	16.76	16.76	12
	18.4											
30	18.4	16.76	16.76	11.6	13.39	13.39	23.33	20.09	18.4	16.76		10
45	16.76	20.09	18.4	20.09	16.76	16.76	18.4					7
60	23.33	26.43	24.99	18.4	16.76	13.39	16.76	26.43	13.39	21.65	20.09	13
	21.65	20.09										
75	20.09	26.43	23.33	16.76	20.09	16.76	20.09					7
90	26.43	21.65	26.43	21.65	20.09	18.4	24.99					7
105	28.13	21.65	31.28	21.65	21.65	23.33	26.43					7
120	20.09	28.13	26.43	20.09	23.33	16.76	32.88	32.88	32.88	26.43	29.76	11
135	40.4	37.49	53.83	26.43	16.76	26.43	24.99					7
150	34.35	35.88	43.54	35.88	16.76	21.65	24.99					7
165	34.35	37.49	20.09	31.28	24.99	34.35	38.92					7
180	35.88	38.92	32.88	32.88	28.13	29.76	35.88	35.88	41.94	31.28		10
195	41.94	41.94	44.92	29.76	28.13	34.35	38.92					7
210	40.4	46.64	58.38	29.76	41.94	37.49	29.76					7
225	41.94	49.33	69.96	41.94	29.76	32.88						6
N=17												177

Table S4: Mature RNA species copy numbers of the PPARδ ligand induction experiments. Calculated copy numbers of the experiments reported in Fig. 2B (mature mRNA, observable 2) are shown. Time t is in minutes, n refers to the number of replica data points at the same time point and N is the number of time points examined.

t	pre-mRNA							n
0	4.61	3.44	3.5	6.66	10.1	11.34	7.22	7
1	8.89	6.41	3.98					3
2	7.02	9.86	3.93					3
3	5.55	7.45	9.22					3
4	3.93	4.17	10.57					3
5	1.82	0.91	1.82	4.03	5.75	4.14		6
6	3.87	7.17	3.39					3
7	1.7	0.98	2.05	3.11	3.71	3.39		6
8	2.39	5.19	6.61	1.34	1.58	0.85		6
9	5.8	4.61	5.08	1.64	1.88	1.7		6
10	0.66	1.16	2.62	1.04	2.73	1.28		6
15	0.52	0.98	0.66	0.52				4
N=12								56
	**mature mRNA**							
0	38.8	30.87	51.24	36.38	35.77	28.39	46.54	7
10	29.01	41.79	25.25					3
20	33.94	32.1	29.01					3
30	35.16	35.16	47.72					3
45	31.49	48.31						2
50	30.25	44.18						2
60	29.63	22.07	36.98	27.77	36.38			5
90	28.39	18.19	33.94					3
120	30.87	15.54	29.01					3
150	29.01	12.85	30.87					3
180	18.84	12.17	22.71					3
N=11								37

Table S5: RNA species copy numbers in the RNA decay experiments. Calculated copy numbers of the experiments reported in Fig. 2C (pre-RNA, observable 1) and Fig. 2D (mature mRNA, observable 2) are shown. Time t is in minutes, n refers to the number of replica data points at the same time point and N is the number of time points examined.

Model	k_{act}/k_{tf}	k_{dea}/k_{pol}	k_{rev}	k_{ini}	k_{elo}	k_{spl}	k_{mat}	k_{deg}	$x_0^{spl/pro}$
n=0	-	-	-	0.1887	-	-	0.0797	0.0038	2.5959
n=2	0.5008	0.7248		1.0097			0.0984	0.005	2.7671
n=1	0.0087	0.0251	0.1220	1.7523	0.3906	0.1699	0.0221	0.0087	2.7640
n=3	0.0382	0.0260	0.2153	0.8114	2.8624	0.5034	0.4122	0.0260	2.7585
n=5	0.1061	1.3034	1.1455	3.4361	2.2717	0.5116	0.1607	0.0247	2.6614
n=10	0.6509	2.0463	2.5940	1.1386	3.2848	0.9878	0.2382	0.0473	2.4357
n=20	1.2009	6.9756	5.0111	1.7545	7.7699	1.8565	0.5549	0.0875	2.5040

Table S6: Best fitting parameters. These parameters are used for the models n = 0, n=2, n = 1, n = 3, n = 5, n = 10, n = 20; k_{tf} and k_{pol} are indicated for model n = 2.

model	$V(\hat{p})/N$	$V^{l,pR}(\hat{p})/N^{l,pR}$	$V^{l,mR}(\hat{p})/N^{l,mR}$	$V^{d,pR}(\hat{p})/N^{d,pR}$	run test	AIC
n=0	0.49	0.42	0.45	0.78	2.09	0.51
n=2	0.48	0.43	0.45	0.74	1.67	0.51
n=1	0.42	0.36	0.39	0.7	0.83	0.47
n=3	0.43	0.4	0.38	0.7	0.63	0.48
n=5	0.44	0.41	0.38	0.7	1.04	0.48
n=10	0.44	0.42	0.38	0.73	1.04	0.49
n=20	0.44	0.42	0.38	0.72	1.46	0.49

Table S7: Results of the residual analysis for models n = 0-20.

	k_{act}	k_{dea}	k_{rev}	k_{ini}	k_{elo}	k_{spl}	k_{mat}	k_{deg}
k_{act}	1.000	-0.925	0.817	-0.942	0.087	-0.106	0.167	0.004
k_{dea}	-0.925	1.000	-0.736	0.998	-0.089	0.078	-0.166	-0.005
k_{rev}	0.817	-0.736	1.000	-0.771	0.076	-0.063	0.236	0.071
k_{ini}	-0.942	0.998	-0.771	1.000	-0.112	0.108	-0.193	0.018
k_{elo}	0.087	-0.089	0.076	-0.112	1.000	-0.789	0.548	-0.724
k_{spl}	-0.106	0.078	-0.063	0.108	-0.789	1.000	-0.695	0.918
k_{mat}	0.167	-0.166	0.236	-0.193	0.548	-0.695	1.000	-0.459
k_{deg}	0.004	-0.005	0.071	0.018	-0.724	0.918	-0.459	1.000

Table S8: Pairwise covariance coefficients of the parameters in the n = 5 model.

**t**	**pre-mRNA**				**n**
0	3.05	4.01	2.74	3.81	4
15	2.94	3.43	2.15	5.57	4
30	4.65	3.22	3.33		3
45	3.33	4.57	3.81		3
60	4.08	4.65	3.43	4.74	4
75	4.29	5.4	2.85	6.55	4
90	4.19	5.3	2.94	7.02	4
105	4.29	4.37	2.54	5.01	4
120	3.91	3.22	4.74		3
135	5.21	2.64	5.3		3
150	4.74	6.39	3.33	5.21	4
165	4.29	6.39	3.22	4.65	4
180	3.72	5.3	3.43	5.93	4
195	5.3	2.94	4.29	7.11	4
210	4.46	5.75	5.57	7.28	4
N=16					56
	**mature mRNA**				
0	13.39	11.6	16.76	21.65	4
15	8.08	8.08	16.76	15.07	4
30	16.76	16.76	15.07	8.08	4
45	13.39	9.86	16.76	15.07	4
60	18.4	15.07	15.07	9.86	4
75	15.07	13.39	16.76	9.86	4
90	20.09	21.65	23.33		3
105	11.6	18.4	21.65		3
120	11.6	23.33	16.76		3
135	18.4	15.07	18.4		3
150	23.33	29.76	18.4		3
165	15.07	6.21	16.76	38.92	4
180	13.39	15.07	15.07	24.99	4
195	13.39	18.4	35.88	31.28	4
210	11.6	23.33	21.65	18.4	4
N=16					55

Table S9: RNA abundance data after splicing inhibition. Copy numbers are indicated.

	k_{act}	k_{dea}	k_{rev}	k_{ini}	k_{elo}	k_{spl}	k_{mat}	k_{deg}
$V^s(\hat{p})/N^s$ run 1	6.64	6.64	6.71	6.64	6.74	1.47	6.71	6.61
a run 1	1.1288	0.8965	4.0000	1.1119	4.0000	0.2650	0.3526	1.1650
$V^s(\hat{p})/N^s$ run 2	1.44	1.44	1.43	1.44	1.47		1.46	1.39
a run 2	0.9546	1.0467	0.3545	0.9565	4.0000		0.5324	1.1257

Table S10: Residuals and parameter adjustment factor for data re-fitting after splicing inhibition. $V^s(\hat{p})/N^s$ is the object function divided by the number of data points, a is the ratio between the new parameter and the old one.

References

1. Draper, N.R., and Smith, H. (1988). John Wiley&Sons, Inc, New York.
2. Price, W.L. (1983) Global optimization by controlled random search. *Journal of Optimization Theory and Applications*, **40**, 333-348.
3. Analytical_Methods_Committee. (2001), *AMC technical brief*. Royal Society of Chemistry, Vol. 6.
